# A novel higher performance nomogram based on explainable machine learning for predicting mortality risk in stroke patients within 30 days based on clinical features on the first day ICU admission

**DOI:** 10.1186/s12911-024-02547-7

**Published:** 2024-06-07

**Authors:** Haoran Chen, Fengchun Yang, Yifan Duan, Lin Yang, Jiao Li

**Affiliations:** 1 Institute of Medical Information/Library, Chinese Academy of Medical Sciences & Peking Union Medical College, Beijing, 100020 China; 2 Key Laboratory of Medical Information Intelligent Technology, Chinese Academy of Medical Sciences, Beijing, 100020 China

**Keywords:** Stroke, Explainable machine learning, Nomogram, Prognostic model, MIMIC database

## Abstract

**Background:**

This study aimed to develop a higher performance nomogram based on explainable machine learning methods, and to predict the risk of death of stroke patients within 30 days based on clinical characteristics on the first day of intensive care units (ICU) admission.

**Methods:**

Data relating to stroke patients were extracted from the Medical Information Marketplace of the Intensive Care (MIMIC) IV and III database. The LightGBM machine learning approach together with Shapely additive explanations (termed as explain machine learning, EML) was used to select clinical features and define cut-off points for the selected features. These selected features and cut-off points were then evaluated using the Cox proportional hazards regression model and Kaplan-Meier survival curves. Finally, logistic regression-based nomograms for predicting 30-day mortality of stroke patients were constructed using original variables and variables dichotomized by cut-off points, respectively. The performance of two nomograms were evaluated in overall and individual dimension.

**Results:**

A total of 2982 stroke patients and 64 clinical features were included, and the 30-day mortality rate was 23.6% in the MIMIC-IV datasets. 10 variables (“sofa (sepsis-related organ failure assessment)”, “minimum glucose”, “maximum sodium”, “age”, “mean spo2 (blood oxygen saturation)”, “maximum temperature”, “maximum heart rate”, “minimum bun (blood urea nitrogen)”, “minimum wbc (white blood cells)” and “charlson comorbidity index”) and respective cut-off points were defined from the EML. In the Cox proportional hazards regression model (Cox regression) and Kaplan-Meier survival curves, after grouping stroke patients according to the cut-off point of each variable, patients belonging to the high-risk subgroup were associated with higher 30-day mortality than those in the low-risk subgroup. The evaluation of nomograms found that the EML-based nomogram not only outperformed the conventional nomogram in NIR (net reclassification index), brier score and clinical net benefits in overall dimension, but also significant improved in individual dimension especially for low “maximum temperature” patients.

**Conclusions:**

The 10 selected first-day ICU admission clinical features require greater attention for stroke patients. And the nomogram based on explainable machine learning will have greater clinical application.

**Supplementary Information:**

The online version contains supplementary material available at 10.1186/s12911-024-02547-7.

## Introduction

The 2016 Global Burden of Disease Study showed that nearly a quarter of people were at risk of developing stroke in their lifetime, and updated data from the Global Survey to 2019 showed that stroke remained the second leading cause of death worldwide, with the absolute number of new cases increasing by 70% from 1990 to 2019 [1]. In addition, an increasing number of stroke patients are being admitted to intensive care units (ICU), but with high mortality and other poor functional outcomes [2, 3]. Monitoring information during ICU was important to improve patient care and prognosis [4, 5], and several studies have shown that changes in vital signs and laboratory indictors always precede the rapid deterioration of a patient’s condition [6, 7]. Similarly, the prognosis of stroke patients admitted to ICU was strongly influenced by the monitoring of various clinical features [8, 9]. Jun Zhao et al. found that abnormalities in inflammatory biomarkers such as neutrophil to lymphocyte ratio and platelet to lymphocyte ratio were associated with increased stroke mortality [10]. At the same time, monitoring the short-term mortality of ICU stroke patients was of greater significance. A clinical cohort study from the Dutch National Intensive Care Database found a higher short-term (within 30 days) mortality rate for stroke patients admitted to the ICU out of a total of 7046 stroke patients, but their mortality rate stabilized after 30 days in the ICU [11].

Nomogram, a visual representation of complex mathematical formulas, is increasingly used in clinical decision support and personalized medicine due to its simplicity and straightforward [12]. And medical nomograms can use biological and clinical variables to determine the prognosis of a specific patient [13, 14]. Nomograms were always developed by logistic and Cox regression models in previous studies, however, these models were based on linear assumptions and they can’t handle the non-linear relationships in clinical practice [15–17]. In contrast, machine learning models can handle non-linear relationships in real-world settings and exhibit better accuracy: a study found the machine learning models outperformed linear regression models (logistic and Cox models) in predicting the risk of death of cervical cancer [17]. But the ‘black-box’ property of machine learning to clinicians limited its clinical applicability compared to nomogram [18]. We therefore aimed to combine the strengths of nomogram and machine learning to develop higher performance and easier to use clinical prediction nomogram.

The purpose of this study was to develop a nomogram based on explainable machine learning to predict the risk of death in stroke patients within 30 days using available clinical data from the first day of ICU admission.

## Materials and methods

### Study design

**Fig. 1:**
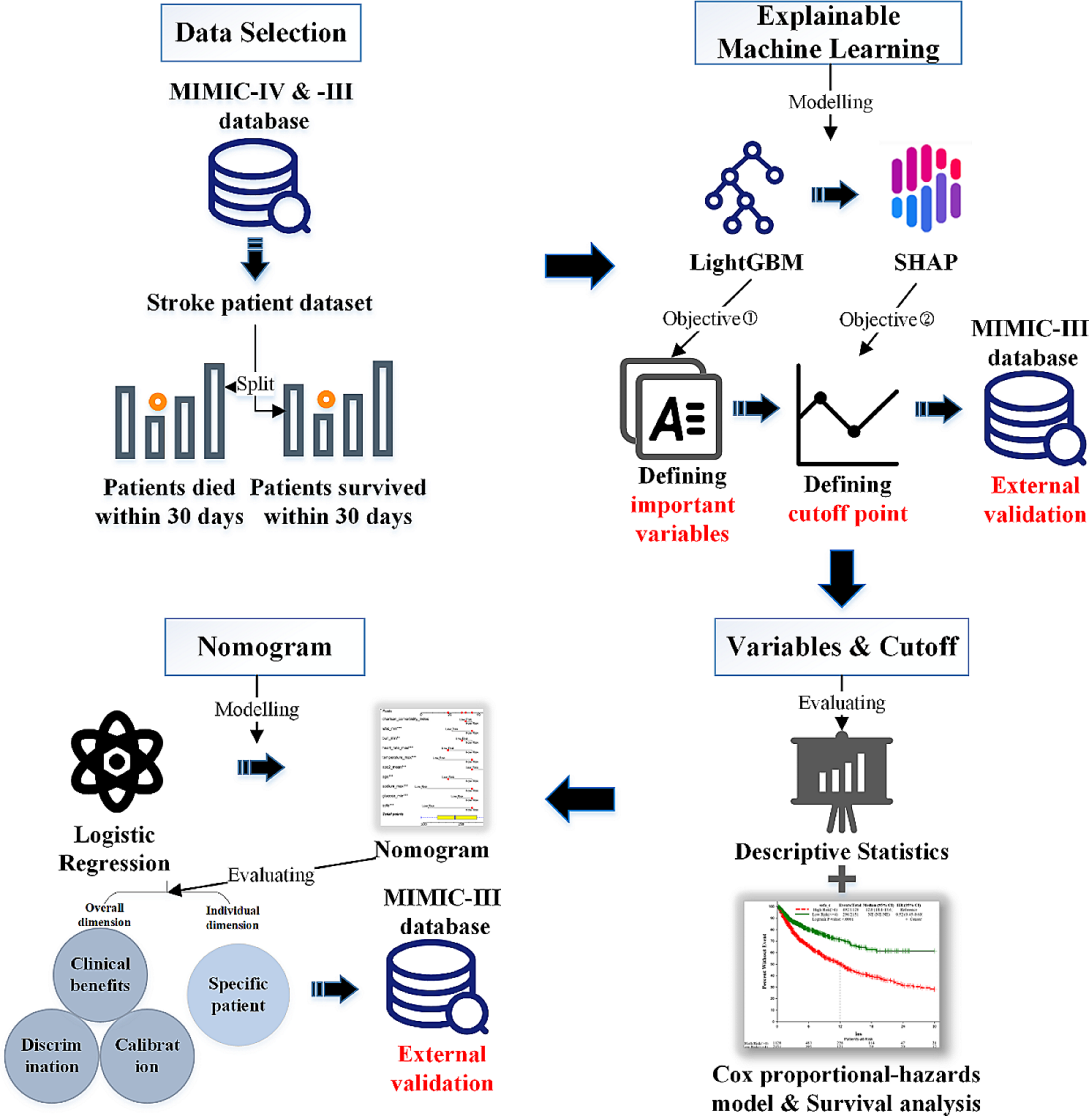
Study design of our research. SHAP: Shapely additive explanations

The design of this study consisted of four stages (Fig. 1): (1) access to the MIMIC-IV and MIMIC-III database and to select suitable stroke patients and associated clinical features; (2) development and validation of explainable machine learning; (3) evaluation of selected variables and cut-off points; (4) construction and evaluation of nomogram.

### Data selection

All data for this study were retrieved from the MIMIC-IV (version 1.0) and MIMIC-III (version 1.4) database. MIMIC-IV was a contemporary electronic health record datasets and provided clinical data on intensive-care for patients admitted to hospital between 2008 and 2019 [19], MIMIC-III comprised health-related data associated with patients between 2001 and 2012 [20]. The data of MIMIC was de-identified and informed consent was waived by the institutional Review Board at the Beth Israel Deaconess Medical Center. After passing the Human Subject Research Course (certification number: 11,467,961), we accessed the MIMIC database and extracted the clinical data of stroke patients. The ninth and tenth editions of the International Classification of Diseases code were used to identify stroke patients, referring to previous study [21]. The following inclusion criteria were further to screen for suitable stroke patients: (1) age between 18 and 89 years old (all patients’ age older than 89 years were not accurate); (2) only patients with one stay_id were included (excluding patients with multiple ICU admissions from the same hospital admission); (3) the length of ICU stay was less than 30 days. The detailed processes of stroke patients selection was shown in Figure S1.

We used Structured Query Language (SQL) with PostgreSQL (version 13.11) and Navicat Premium (version 16.0.11) to extract data on stroke patients, as well as many clinical features such age, gender and ethnicity. And we further extracted laboratory measurements, comorbidities, vital signs and disease severity assessment within first day of the patient’s admission to the ICU (e.g., first day urine output, first day blood gas). The type of stroke diagnosis (including ischemic stroke, transient ischemic attack (TIA), subarachnoid hemorrhage and intracerebral hemorrhage) was also included as an important feature for the prognosis of stroke patients. Table S1 detailed the total of 64 relevant features extracted in this study.

### Developing and validating explainable machine learning

The LightGBM was used as a machine learning algorithm in this study to predict the risk of mortality within 30-day in ICU stroke patients. The LightGBM was an innovative tree-based ensemble learning algorithm and was characterized by fasting speed, high predictive accuracy and less memory usage by the Gradient-based One-side Sample and Exclusive Feature Bundling [22]. The MIMIC-IV datasets was randomly split into training datasets (80%) and testing datasets (20%). We searched for the best-performing combination of parameters for LightGBM by the method of Bayesian optimization with the objective of maximizing the area under the receiver operating characteristics curve (AUC) in the testing datasets. And the quality of the optimized model was assessed based on 5-fold cross-validation approach. In addition, we applied the Shapely additive explanations (SHAP) to explain the output of the LightGBM. SHAP was a novel model interpretation method from coalitional game theory that can examine effects of each variable on the output of the machine learning by SHAP values [18]. Especially, SHAP summary plots were used to determine the feature importance and thus select suitable variables, and SHAP partial dependency plots (PDPs) were used to find the cut-off point for the selected variables [23, 24]. To further validate the robustness of the selected important features and cut-off points, we evaluated the trained LightGBM model in an external validation dataset (MIMIC-III, *n* = 2252) [20] and used SHAP to interpret it.

### Evaluating selected variables and found cut-off points

For the convenience of nomogram development and ease of clinical application, we selected the top 10 variables as ranked by the SHAP summary plot. In order to assess the ability of the selected 10 variables to discriminate two groups (death/survival), we employed the kruskal-wallis rank sum test to compare the difference between the two groups for each selected variable.

Based on the found cut-off point of each variable, all 10 otherwise continuous variables were dichotomized into categorical variables, so the stroke patients were stratified into two subgroups (high risk and low risk). We have subsequently evaluated the performance of those found cut-off points by the following three methods: (1) The chi-square test was used to compare statistical distribution of these categorical variables between death and survival groups; (2) the Cox regression model was performed to determine the association between each selected variables and 30-day mortality in stroke patients, using the high-risk subgroup as the reference; (3) the Kaplan-Meier (K-M) survival curve followed by log-rank test was utilized to compare the difference in ICU survival rate within 30 days between two subgroups based on each selected variable.

### Constructing and evaluating nomogram

Taking survival status of 30-day after ICU admission for stroke patients as the dependent variables, we constructed separate logistic regression-based nomograms for the original 10 continuous and categorical variables (i.e. dichotomous continuous variables) for predicting the risk of death in ICU for stroke patients. Discriminatory power (AUC and index of net reclassification (NRI)), calibration power (calibration curve and brier score) and clinical applicability (decision curve analysis (DCA)) were used in overall dimension and a specific patient (ID: 2846) was regarded as an example in individual dimension to compare the performance of the two nomograms [12]. The NRI was a proxy for the AUC and was used to assess improvements in risk prediction from a new model [25]. How close the nomogram estimated risk was to the observed risk was assessed by calibration curve and brier score [12, 26]. And the DCA can assess whether nomogram-assisted decisions improve patient outcomes [12]. In addition, two nomograms’ AUC were compared by the DeLong test using 2000 bootstrapped resampling to reduce over-fitting [27].

### Statistical analysis

Since the skewness and kurtosis test determined that all continuous variables exhibited non-normality and thus they were expressed as median and range. The spearman rank correlation method was utilized to assess the pairwise correlations and variance inflation factor (VIF) was used to assess the possible multi-collinearity of 10 selected variables. In addition, categorical variables were presented as numbers and percentages. We used the mode and the median to impute missing values for categorical and continuous variables, respectively. In our study, machine learning models were developed and evaluated based on Python (version 3.8.8) from *scikit-learn* (version 1.3.2), *lightgbm* (version 3.3.2), *shap* (version 0.41.0) and *hyperopt* (version 0.2.7); the K-M survival curves and Cox regression models were conducted and plotted by SAS (Statistics Analysis System, version 9.4); other data analyses, development, evaluation and visualization of nomograms were performed using R (version 4.2.1) from *tidyverse* (version 1.3.0), *rms* (version 6.2.0), *pROC* (version 1.18.0), *regplot* (version 1.1), *nricens* (version 1.6), *rmda* (version 1.6), *ggplot2* (version 3.3.6) and *corrplot* (version 0.92). *P* < 0.05 (two-tailed) was considered statistically significant.

## Results

### Statistical description of clinical features

As was shown in Table S2, a total of 2982 ICU patients with stroke were enrolled from MIMIC-IV database, those patients’ mean age was 65.3 years and 48.2% was female, and up to 704 (23.6%) patients died within 30 days. All variables except sex, maximum mbp (mean arterial pressure), minimum ptt (activated partial thromboplatin time), maximum ptt, minimum potassium, minimum chloride and minimum sodium were significantly different in survived and dead stroke patients. For example, the average length of stay (los) of survived patients was higher than those dead counterparts. And the variable with the highest rate of missingness was ‘marital status’ (14.8%). Figure S2 showed that there was no strong correlation between the 10 selected variables (all spearman correlation coefficient < 0.5), and the VIF of 10 selected variables less than 4 (Table S3), so we assumed that there was no mulit-collinearity between them. Therefore all 10 selected variables were included in the nomogram construction.

### Explainable machine learning

**Fig. 2:**
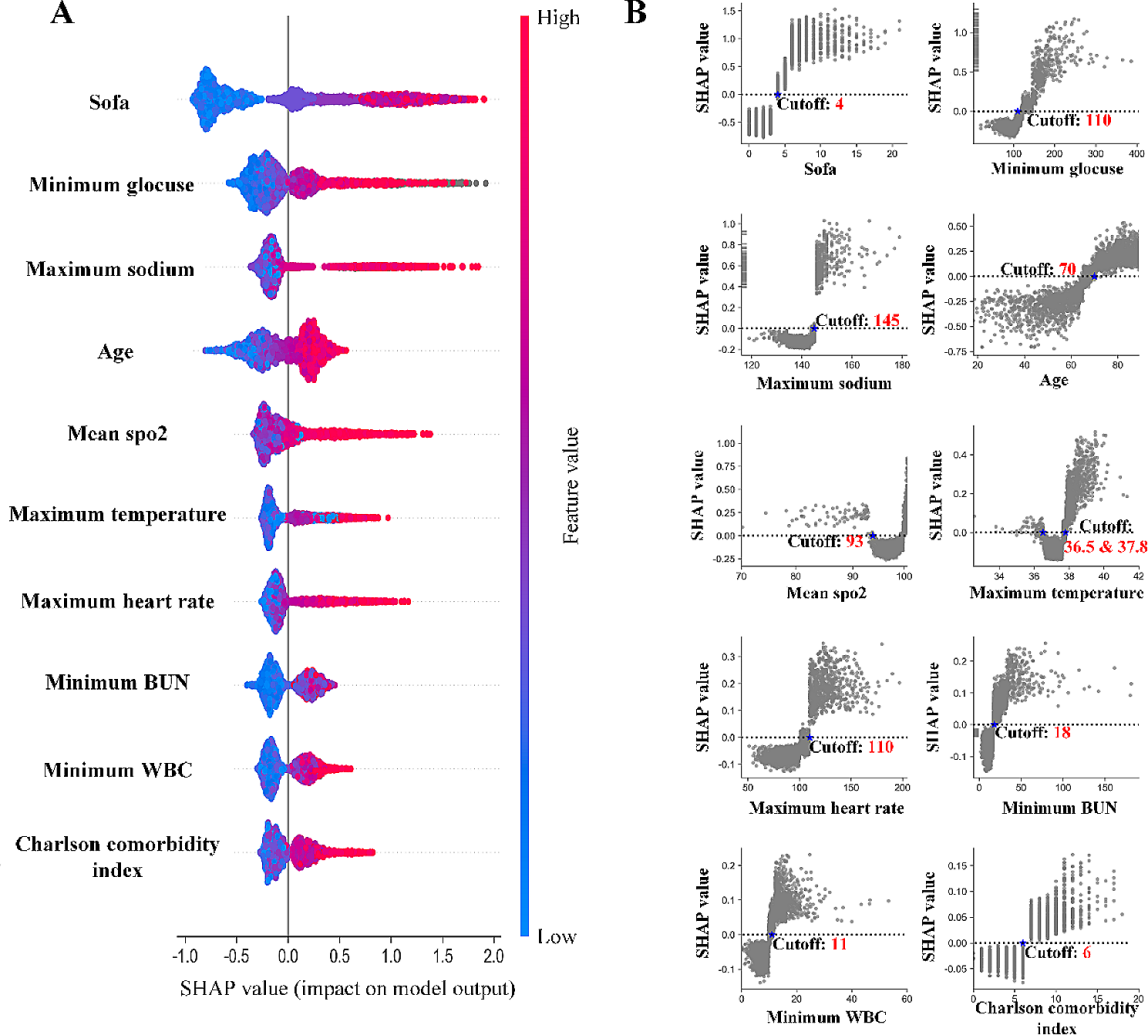
Explainable LightGBM results of using the shapely additive explanations (SHAP) in the testing datasets. **A**: the SHAP feature analysis summary plot of the top 10 variables. The X-axis is for the SHAP value and Y-axis is for feature, ranked in descending order for feature importance. Each dot in the figure is the SHAP value of a patient at specific feature value, and red represents higher feature values for positive influence on death risk, but blue represents the opposite effect. **B**: the SHAP partial dependency plots (PDPs) for each selected variable. The X-axis is for each feature and the Y-axis is for the SHAP values. SHAP values greater than 0 indicate that the feature at this specific value is a risk factor for death. The cut-off point was the point where the SHAP value was equal to zero

Figure S3.A & B showed the LightGBM performed well (AUC: 0.88 ± 0.01, sensitivity: 0.809 and specificity: 0.809) in predicting the risk of death in stroke patients. The SHAP summary plot ranked features in descending order (from top to bottom) of importance, and we observed that the “sofa” was the most important variables for the prediction capability of the LightGBM (Fig. 2.A). The other top nine variables selected were “minimum glucose”, “maximum sodium”, “age”, “mean spo2”, “maximum temperature”, “maximum heart rate”, “minimum bun”, “minimum wbc” and “charlson comorbidity index”, respectively. The SHAP PDPs (Fig. 2.B) revealed that each selected feature can impact different effects on the death risk of stroke patients at various feature values, and the SHAP values (Y-axis) changed on both sides of the cut-off point. The “sofa”, for example, generally had a SHAP value greater than 0 when the sofa was > 4, indicating that high sofa values had a significant negative impact on the survival of stroke patients. The cut-off points for the other 9 variables were: “minimum glucose”, 100; “maximum sodium”, 145; “age”, 70; “mean spo2”, 93; “maximum temperature”, 36.5 and 37.8; “maximum heart rate”, 100; “minimum bun”, 18; “minimum wbc”, 11; “charlson comorbidity index”, 6. In the external validation analysis, the performance of the trained LightGBM was good (AUC: 0.84 ± 0.01, sensitivity: 0.772 and specificity: 0.791, Figure S4). For the 10 important features defined from the MIMIC-IV datasets, their overall impact on mortality risk didn’t change (distribution of SHAP values) in the MIMIC-III datasets, but the importance rankings of “sofa” and “charlson comorbidity index” changed considerably (Figure S5.A); and Figure S5.B similarly revealed that the cut-off points found from the external validation set were very close to those of the testing set, except for the cut-off points corresponding to the “sofa” and “charlson comorbidity index”, and in particular, the cut-off points for features such as minimum glucose, mean spo2, maximum temperature, minimum bun and minimum wbc were identical in the two datasets.

### Evaluation of selected variables and cut-off points

Table 1 showed the results of the statistical descriptions for the selected 10 variables, showing significant differences between survived and dead stroke patients on all 10 variables (*P* < 0.001), thus indicating that the 10 selected variables differentiated well between survived and dead stroke patients.

**Table 1:** Comparison of 10 selected variables between survived and dead patients

Variable	Death of Stroke			
	No (*N* = 2278)	Yes (*N* = 704)	Total (*N* = 2982)	*P* value
**sofa**				< .001^1^
Median	3.0	5.5	3.0	
Range	0.0, 17.0	0.0, 21.0	0.0, 21.0	
**minimum glucose**				< .001^1^
Median	105.0	123.0	109.0	
Range	32.0, 283.0	20.0, 365.0	20.0, 365.0	
**maximum sodium**				< .001^1^
Median	141.0	142.0	141.0	
Range	119.0, 174.0	125.0, 179.0	119.0, 179.0	
**age**				< .001^1^
Median	66.7	72.6	68.3	
Range	18.0, 89.0	22.8, 89.0	18.0, 89.0	
**mean spo2**				< .0001^1^
Median	97.1	98.0	97.3	
Range	79.6, 100.0	67.0, 100.0	67.0, 100.0	
**maximum temperature**				< .001^1^
Median	37.3	37.7	37.3	
Range	33.8, 40.2	31.8, 41.3	31.8, 41.3	
**maximum heart rate**				< .001^1^
Median	94.0	105.0	97.0	
Range	55.0, 190.0	56.0, 197.0	55.0, 197.0	
**minimum bun**				< .001^1^
Median	14.0	18.0	15.0	
Range	2.0, 180.0	3.0, 181.0	2.0, 181.0	
**Minimum wbc**				< .001^1^
Median	9.1	10.9	9.4	
Range	0.3, 121.0	0.1, 199.3	0.1, 199.3	
**charlson comorbidity index**				< .001^1^
Median	6.0	7.0	6.0	
Range	0.0, 18.0	1.0, 17.0	0.0, 18.0	

^1^Kruskal-Wallis *p*-value;

Abbreviations: sofa: sepsis-related organ failure assessment; spo2: minimum blood oxygen saturation; bun: blood urea nitrogen; wbc: white blood cells

**Fig. 3:**
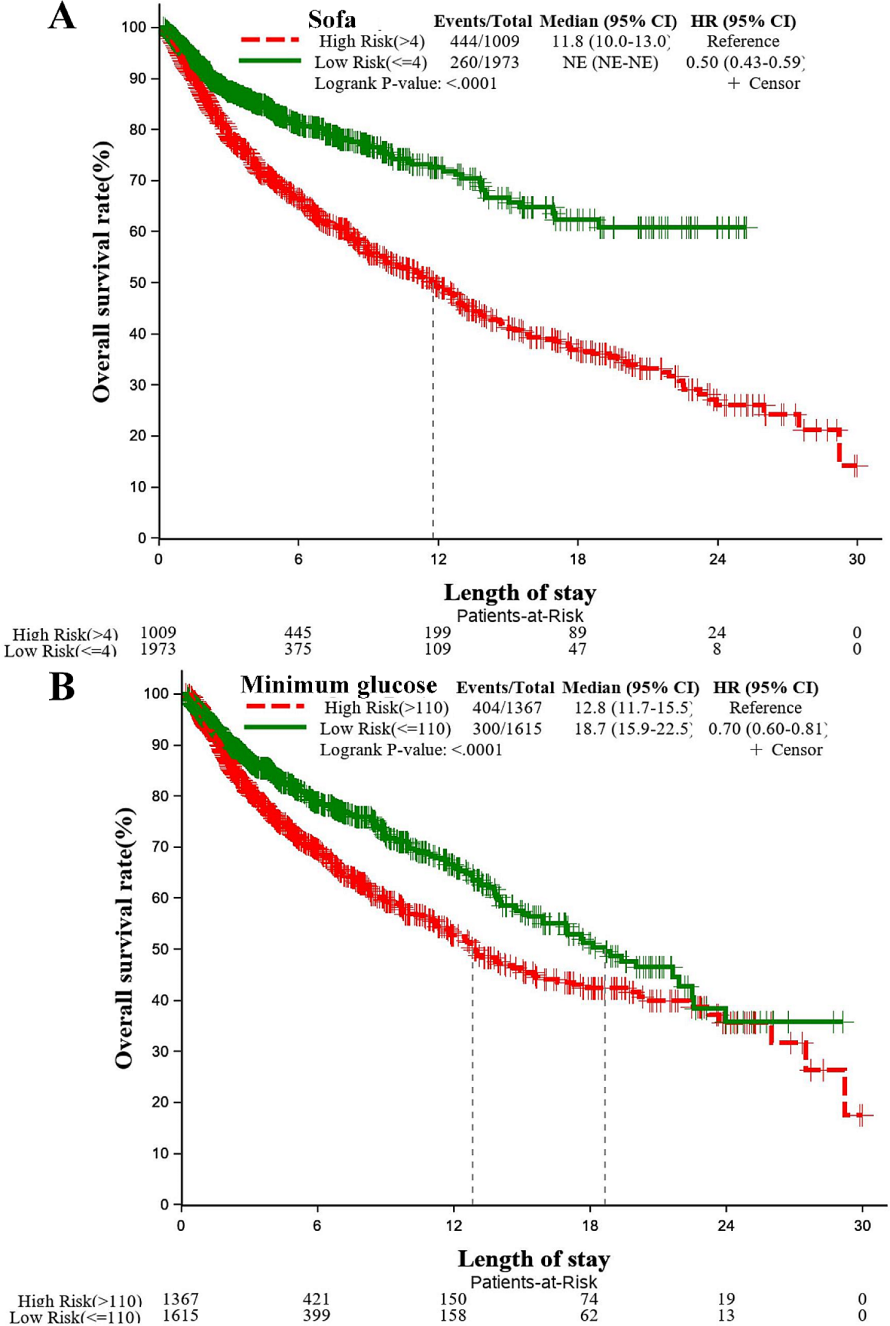
Association between sofa/minimum glucose and death risk of stroke patients by Cox proportional hazards regression models and Kaplan-Meier survival curves using high risk subgroup as the reference. **A**: sofa; **B**: minimum glucose. NE: missing values. HR: hazard ratio

The results of the following three evaluation methods demonstrated that the selected variables’ cut-off points had sufficient discriminatory power to distinguish between survival and death in stroke patients:

1. All categorical variables were shown by χ^2^ test to be statistically different between the two groups of stroke survivors and deaths (Table S4);
2. As shown in the Kaplan-Meier survival plots (Fig. 3 & Fig. S6), for all selected variables, the 30-day overall survival rate for stroke patients were significant lower in the high-risk subgroup than those in the low-risk subgroup ( logrank *P* < 0.001).
3. For all selected variables, the Cox proportional risk hazards models showed that the low-risk subgroup was associated with lower 30-day mortality when compared with the high-risk subgroup (i.e., sofa: HR = 0.50(0.43,0.59), Fig. 3.A).

### Evaluation of the nomogram

**Fig. 4:**
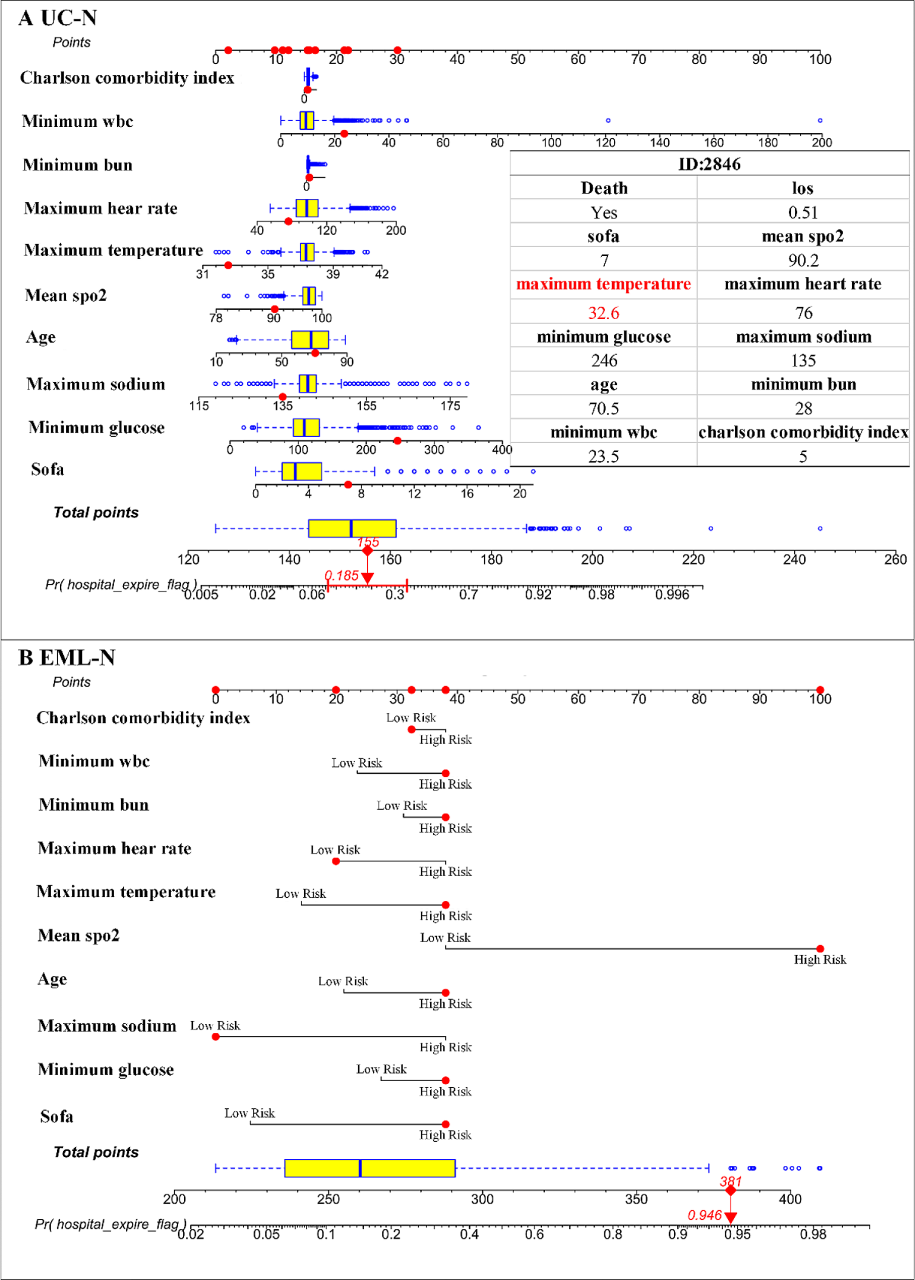
Nomograms for predicting 30-day mortality among stroke patients in the MIMIC-IV datasets. **A**: the nomogram (UC-N) was developed by those selected variables in EML and was continuous; **B**: the nomogram (EML-N) was developed by those dichotomous selected variables in EML and was categorical. UC-N: unchanged nomogram, i.e., the nomogram was developed based on unchanged continuous variables. EML-N: explainable machine learning + nomogram, i.e., the nomogram was developed based on the findings of the EML

The Fig. 4 consisted of two nomograms: one nomogram (Fig. 4.A) used original continuous variables and the other one (Fig. 4.B) used categorical variables. A specific patient can be scored on the basis of the respective features in the two nomograms and assessed for 30-day mortality based on the total score. We compared the performance of the two nomograms through overall evaluation metrics and patient-specific examples, respectively.

#### Overall dimension

**Fig. 5:**
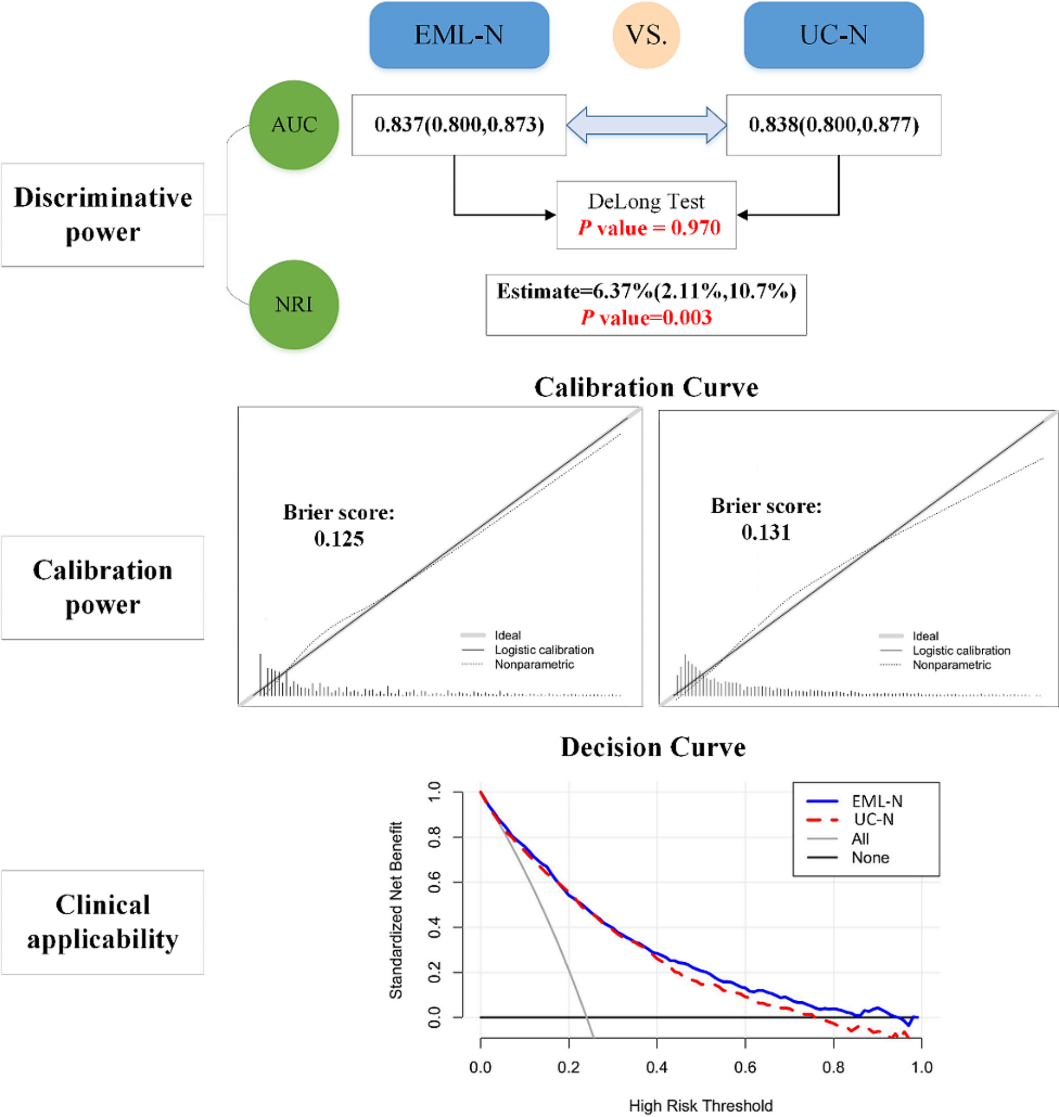
Performance differences in overall dimension of EML-N and UC-N in the MIMIC-IV datasets. EML-N: explainable machine learning + nomogram; UC-N: unchanged nomogram; AUC: area under the receiver operating characteristics curve; NRI: net reclassification index

Figure 5 compared the overall performances of “EML-N” and “UC-N” in the following 3 aspects:

1. Discriminative power: the AUC of EML-N and UC-N was 0.837(0.800, 0.873) and 0.838(0.800, 0.877), and the DeLong test found that there was no significant difference between both nomograms (*P* = 0.970); the NRI of the EML-N had a statistically positive improvement in predicting 30-day mortality compared to the UC-N (6.37% (2.11%, 10.7%)) (*P* < 0.05).
2. Calibration power: the calibration curve of both nomograms all showed that the actual 30-day mortality of stroke patients was consistent with the 30-day mortality predicted, but the Brier score of UC-N was higher than that of the EML-N.
3. Clinical applicability: At higher threshold probability (0.4–0.8), the EML-N had a higher clinical net benefit than UC-N.
4. “EML-N” based on the external validation datasets (MIMIC-III) was not worse than “UC-N” in the above 3 aspects (Figure S7).

#### Individual dimension

Figure 4.A showed the details of a patient with ID 2486 who was in the ICU for only 0.51 days. On her/she first day in the ICU, the “sofa” was 7, “mean spo2” was 90.2, “maximum temperature” was 32.6,” maximum heart rate” was 76, “minimum glucose” was 246, “maximum sodium” was 135, “age” was 70.5, “minimum bun” was 28 and “minimum wbc” was 23.5 and her/his treatment ended with death in the ICU. According to the score corresponding to each individual feature from the first row (the “Point” axis) in the nomogram (Fig. 4): the total score for this patient was 155 in the UC-N, corresponding to a risk of death within 30-day at the 18.5% level (Fig. 4.A); but in the EML-N (Fig. 4.B), the patient’s total score was 381 and her/his risk of ICU death within 30 days was 94.6%. There was no doubt that the predicted outcome from the EML-N for this patient was correct. We suspected that the large difference between the UC-N and the EML-N in predicting the risk of death for this stroke patient was due to the inconsistency of the score for the “maximum temperature” feature. The UC-N was developed from a logistic regression that suggested a linear correlation between “maximum temperature” and stroke mortality, which ignored the risk of death form lower “maximum temperature” in stroke patients. Moreover, our EML-N was much easier to use than the UC-N in defining the scores of individual features.

## Discussion

In this study, we developed a nomogram based on EML to predict the 30-day risk of death in ICU stroke patients, with higher performance compared to the UC-N. In addition, our findings revealed that the ability of EML to identify important variables and explore complex non-linear associations can improve on the shortcomings of traditional linear models (e.g., logistic regression). Our nomogram therefore can allow clinicians to easily and accurately assess the risk of short-term death for stroke patients on the first day of ICU admission, thereby improving patient treatment and care.

The identification of risk factors for death in stroke patients can improve patient management and enable a more accurate estimate of prognosis. From the SHAP summary plot (Fig. 2.A), we incorporated a total of 10 risk factors into the subsequent nomogram construction, with “sofa” being the variable that had the greatest impact on the LightGBM. Though the “sofa” score originated form a score of sepsis-related organ failure assessment, it had been widely used for routine monitoring of acute morbidity in intensive care units [28, 29]. “Sofa” score was a comprehensive assessment of the state of dysfunction in six aspects of the body. Sofa’s predictive value for early mortality risk in stroke patients has been proven: Wei Qin et al. found that the first day “sofa” score had a good predict effect on the stroke patient’s prognosis [29]. In addition, our study revealed that stroke patients with a “sofa” score of greater than 4 had a higher risk of death (Figs. 2.B and 3.A). A meta-analysis found that average mortality also significantly increased in 30-day sepsis mortality in study populations with higher “sofa” score [30]. This study found that the feature importance of “sofa” showed a large difference between the Fig. 2.A and Figure S5.A, and after examining the statistical distributions of “sofa” and “charlson comorbidity index” in MIMIC-III and MIMIC-IV datasets (Figure S8), it was found that “charlson comorbidity index” was not significantly different between dead and surviving stroke patients and between MIMIC-III and MIMIC-IV datasets, whereas “sofa” was present in more patients with high scores in MIMIC-IV datasets (dead or not), which may be the sofa’s high importance in the MIMIC-IV datasets and low importance in MIMIC-III datasets. The other risk factor worth exploring in this study was “maximum temperature”. Temperature management was particularly important for ICU patients given that even small changes in body temperature can lead to changes in inflammation and immune function and had variety of effects on patient outcomes [31–33]. Our study concluded that for stroke patients, the maximum body temperature between 36.5 and 37.8 on their first day in the ICU would reduce the risk of death within 30 days. A large retrospective cohort of 28,679 Australian and 45,038 New Zealand stroke patients found that their maximum body temperature on the first day of ICU admission was between 37 and 39 degrees with a lower risk of death [34], which was more consistent with our findings. Other risk factors, including “age”, “sodium”, “bun” (blood urea nitrogen) and “heart rate”, were also identified in studies of predicting the risk of death in stroke patients based on the MIMIC datasets [8, 10].

Given the huge burden of disease already caused by stroke: stroke alone was responsible for 6.6 million deaths worldwide, small improvements in the accuracy of prognostic-related prediction models for stroke can have huge benefits [35]. Our EML-N was a significant improvement over the UC-N in terms of both the overall dimension and the individual dimension. We believed that it was the following two major improvements in the method we built on the nomogram that had led to the higher performance of our EML-N. Firstly, linear models (including logistic regression and cox regression) were the most common for develop nomogram [12]. However, those linear models were not appropriate when there was a nonlinear association between predictors and outcomes [36]. Daan et al. reported that the restricted cubic splines regression (a nonlinear modeling methods) outperformed the logistic regression with linear terms when assessing the nonlinear relationship between continuous predictors and outcome [36]. Although some studies had fitted non-linear relationships between predictors and outcomes by using variables with cubic splines in logistic regression, the choice of location and number of knots was strongly influenced by a priori experience [9, 36, 37]. Taking the “maximum temperature” variable in this study as an example, we found that cubic spline regressions (RCS) using 3 knots (10th, 50th and 90th percentiles) and 5 knots (5th, 27.5th, 50th, 72.5th and 95th percentiles) showed significantly different trends in their curves after the “maximum temperature” above 38 degrees (results not reported). Moreover, when a large number of variables were included in the RCS, the workload of selecting the best-fit form of all variables was significant and can easily lead to biased results. On the contrary, the PDPs in this study greatly reduced the difficulty of knots selection in RCS and allowed for the non-linear fitting of multiple variables simultaneously. Secondly, the important variables associated with ICU mortality of stroke patients were easily selected by the SHAP summary plot in our study. A common method in selecting important variables was least absolute shrinkage and selection operator algorithm (lasso) in disease research [38]. Zirui Meng et al. utilized lasso to select the important variable in laboratory examination results [39]. However, the lasso was a linear model and it only select a variable that was linearly related with the outcome, and only one variables could be chosen from a set of highly correlated variables [40]. It was certainly possible that some key variables may not be selected. In our study, both UN-N and EML-N were developed based on the variables selected from the SHAP summary plot and they both had high AUC values.

There were several limitations in the study. Firstly, our nomogram was only constructed and validated by MIMIC (III & IV) datasets, so it may not be generalizable to other settings. Secondly, in order to further improve the usability and convenience of the EML-N, we discretized all continuous variables, which may lead to a loss of some information and thus reduced the performance of the nomogram. Thirdly, the feature importance and cut-off point analyses were only conducted by one machine learning method, which could lead to bias in feature and cut-off point selection. Fourthly, given the need for sample size and the exertion of SHAP-based interpretability capability, this study uses the MIMIC-III dataset as the validation set, which inevitably overlaps with the MIMIC-IV dataset for some of the research subjects. Subsequent related studies that focus on prediction performance may try to use the MIMIC-III Clinical Database CareVue subset. In addition, although TIA was often thought of as a herald to stroke only, our study also included patients who were diagnosed with TIA [41]. A large cohort lasting 66 years found that 30.8% (40) of the 130 stroke patients identified at follow-up had a TIA within 30 days [42]. Finally, patients with TIA only accounted for 4.8% of our study, which would not affect the robustness of our nomogram.

## Conclusions

We analyzed the first-day ICU clinical features of stroke patients and developed a higher performing and easier to use nomogram. Our study demonstrated that the explainable machine learning’s ability in dealing nonlinear relationships between variables can be applied to linear models.

### Electronic supplementary material

Below is the link to the electronic supplementary material.

Supplementary Material 1

## Data Availability

The datasets used in this study are available at the database: https://mimic.mit.edu/.

## Code availability

We have made the source code for our experiments available at https://github.com/CHENJIEN2323/Stroke_nomogram.git to ensure the reproducibility of our results.
