# Supplementary material for: A novel higher performance nomogram based on explainable machine learning for predicting mortality risk in stroke patients within 30 days based on clinical features on the first day ICU admission
Source: BMC Med Inform Decis Mak. 2024 Jun 7;24:161. doi: 10.1186/s12911-024-02547-7 (PMC11161998; doi:10.1186/s12911-024-02547-7)
Supplement: Supplementary file 1 — Supplementary Material 1 [file 12911_2024_2547_MOESM1_ESM.docx]

**A novel higher performance nomogram based on explainable machine learning for predicting mortality risk in stroke patients within 30 days based on clinical features on the first day ICU admission**

Haoran Chen ^1, 2^, Fengchun Yang ^1, 2^, Yifan Duan^1^, Lin Yang^1, 2^and Jiao Li ^1,2^*

|  |
| --- |

^1^ Institute of Medical Information/Library, Chinese Academy of Medical Sciences & Peking Union Medical College, Beijing 100020, China

^2^ Key Laboratory of Medical Information Intelligent Technology, Chinese Academy of Medical Sciences, Beijing 100020, China

***** Correspondence: li.jiao@imicams.ac.cn; Tel: +(86)10-52328740

**Figure S1**: The stroke patient selection processes for data selection from the MIMIC-IV database;

**Figure S2**: Spearman correlation matrix of ten selected variables.

**Figure S3:** The performance of the optimized LightGBM in the testing datasets.

**Figure S4**. The performance of the optimized LightGBM in the external validation datasets (n=2252).

**Figure S5**. Explainable LightGBM results of using the Shapely additive explanations (SHAP) in the external validation datasets.

**Figure S6**: Association between selected variables and death risk of stroke patients by Cox proportional hazards models and Kaplan-Meier (KM) survival curves using high risk subgroup as the reference.

**Figure S7** Performance differences in overall dimension of EML-N and UC-N in the MIMIC-III datasets.

**Figure S8.** The distribution of “sofa” and “charlson comorbidity index” in MIMIC-III and MIMIC-IV datasets.

**Table S1:** All variables extracted from the MIMIC-IV and their explanations in our study.

**Table S2:** Comparison of all variables between survived and dead patients.

**Table S3:** Covariance diagnosis for 10 variables selected.

**Table S4:** Comparison of 10 selected categorical variables between survived and dead patients.


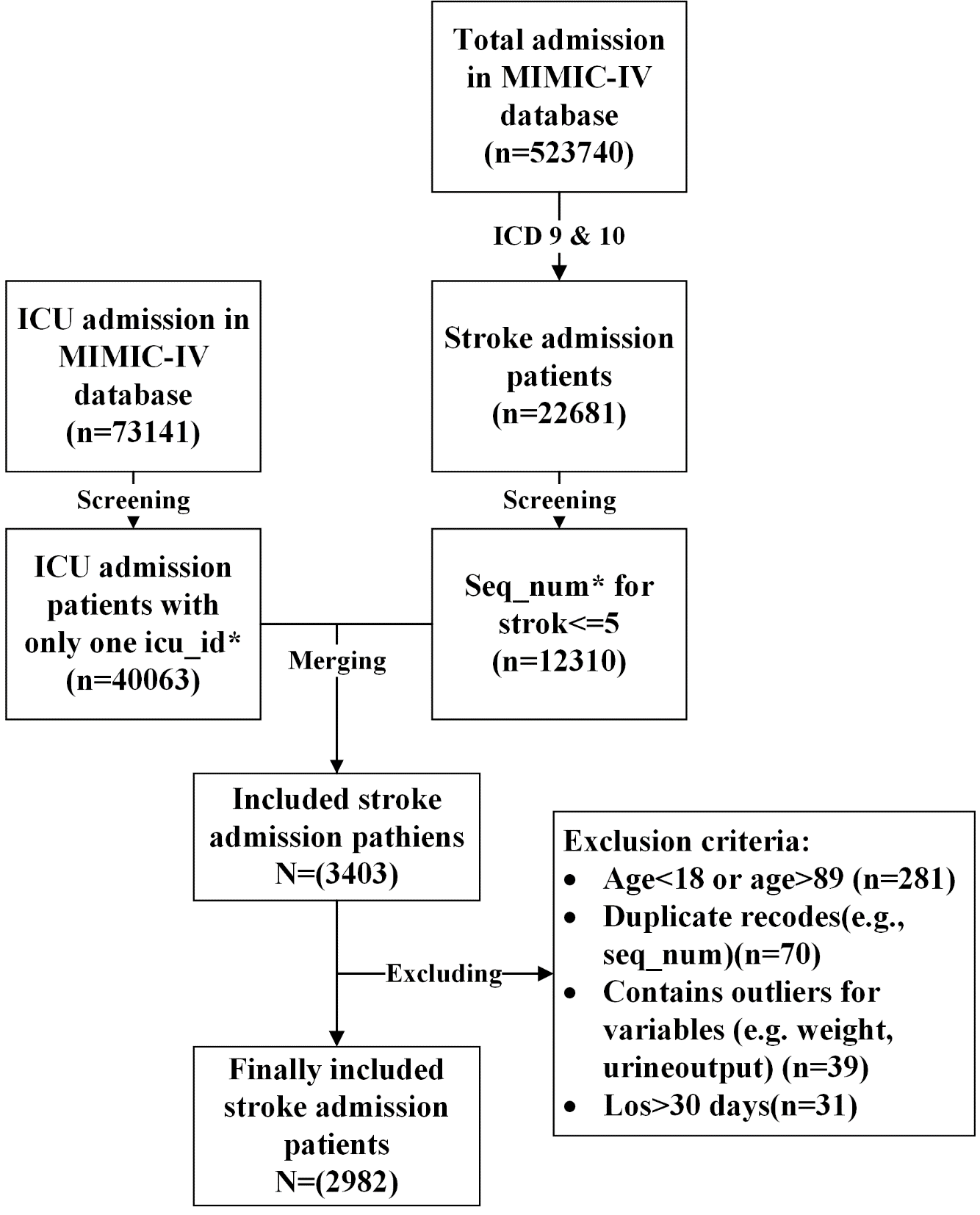


**Figure S1** The stroke patient selection processes for data selection from the MIMIC-IV database.


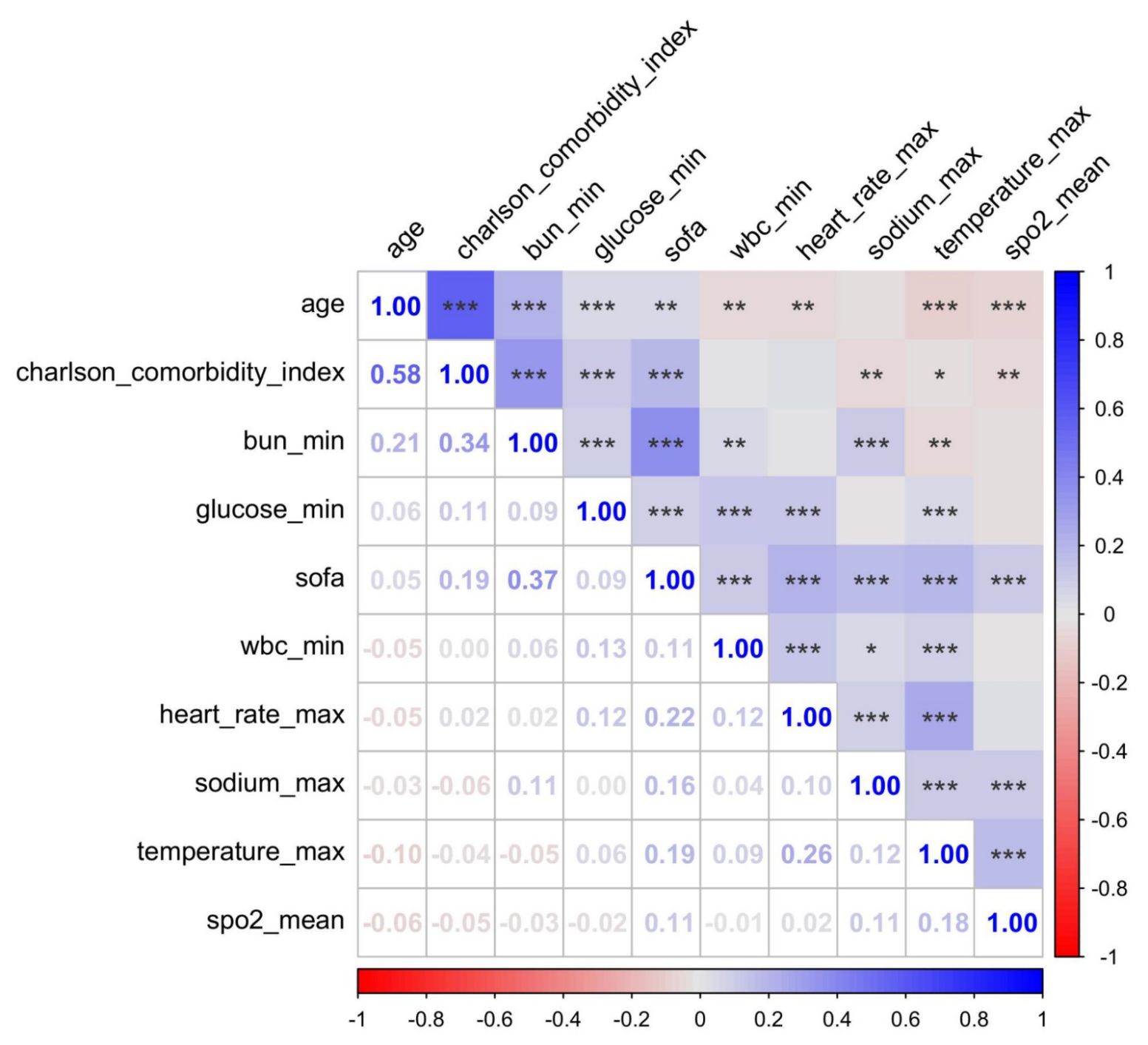


**Figure S2** Spearman correlation matrix of ten selected variables.


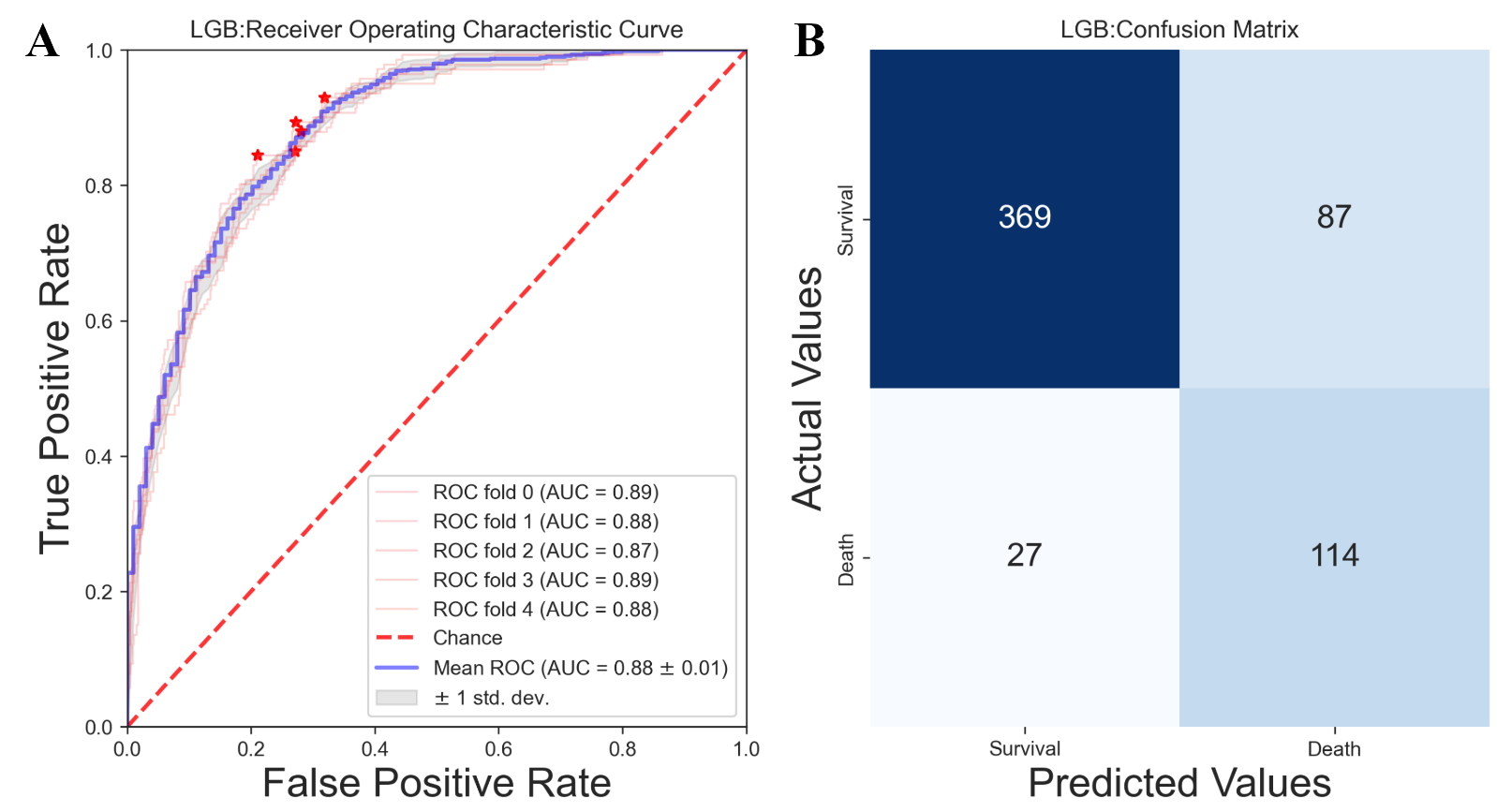


**Figure S3.** The performance of the optimized LightGBM in the testing datasets. A: the operating characteristics curve of 5-fold cross-validation of LightGBM in the datasets. B: the confusion matrix of the LightGBM in the fifth fold datasets. Sensitivity=0.809(114/(27+114)), Specificity=0.809(369/(87+369)). AUC: area under the curve.


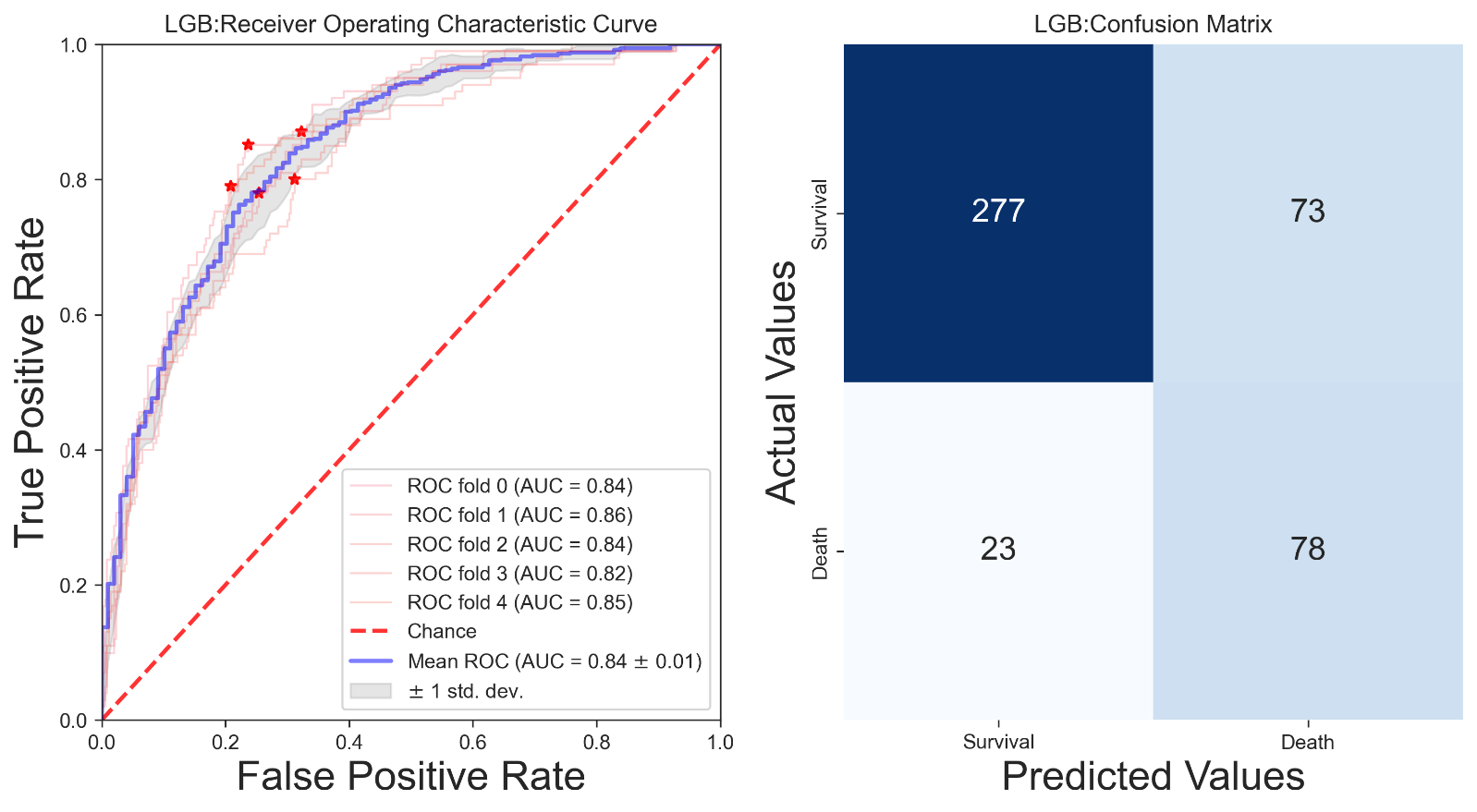
Figure S4. The performance of the optimized LightGBM in the external validation datasets (n=2252). A: the operating characteristics curve of 5-fold cross-validation of LightGBM in the datasets. B: the confusion matrix of the LightGBM in the fifth fold datasets. Sensitivity=0.772(78(23+78)), Specificity=0.791(277/(73+277)). AUC: area under the curve.


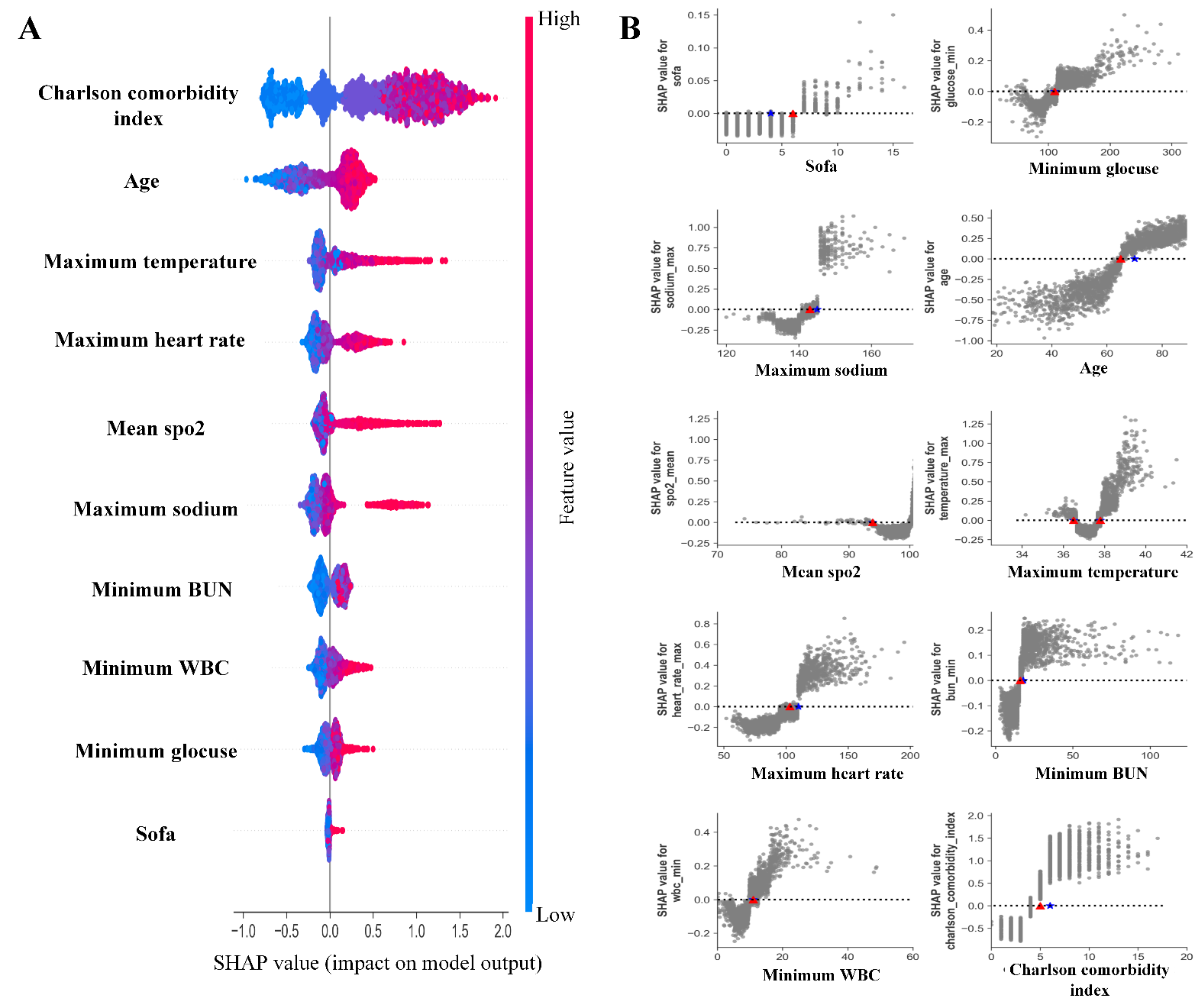


**Figure S5**. Explainable LightGBM results of using the Shapely additive explanations (SHAP) in the external validation datasets. A. The SHAP feature analysis summary plot of the top 10 variables. The X-axis is for the SHAP value and Y-axis is for feature, ranked in descending order for feature importance. Each dot in the figure is the SHAP value of a patient at specific feature value, and red represents higher feature values for positive influence on death risk, but blue represents the opposite effect. B. The SHAP partial dependency plots (PDPs) for each selected variable. The X-axis is for each feature and the Y-axis is for the SHAP values. SHAP values greater than 0 indicate that the feature at this specific value is a risk factor for death. The cutoff point was the point where the SHAP value was equal to zero. The blue “*” meant the cut-off point defined by the MIMIC-IV dataset, the red “△” meant that the cut-off point defined by the MIMIC-III dataset.


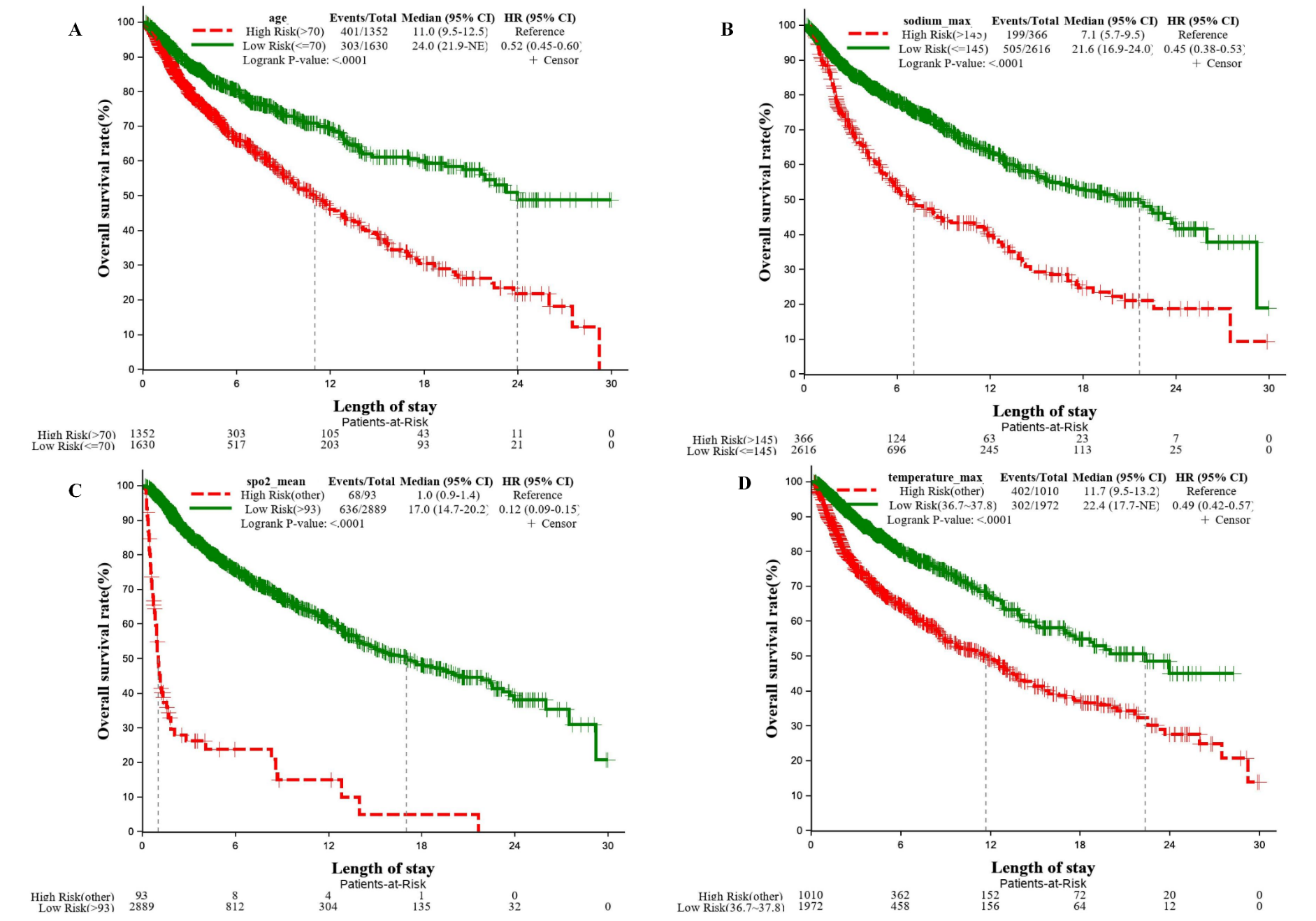


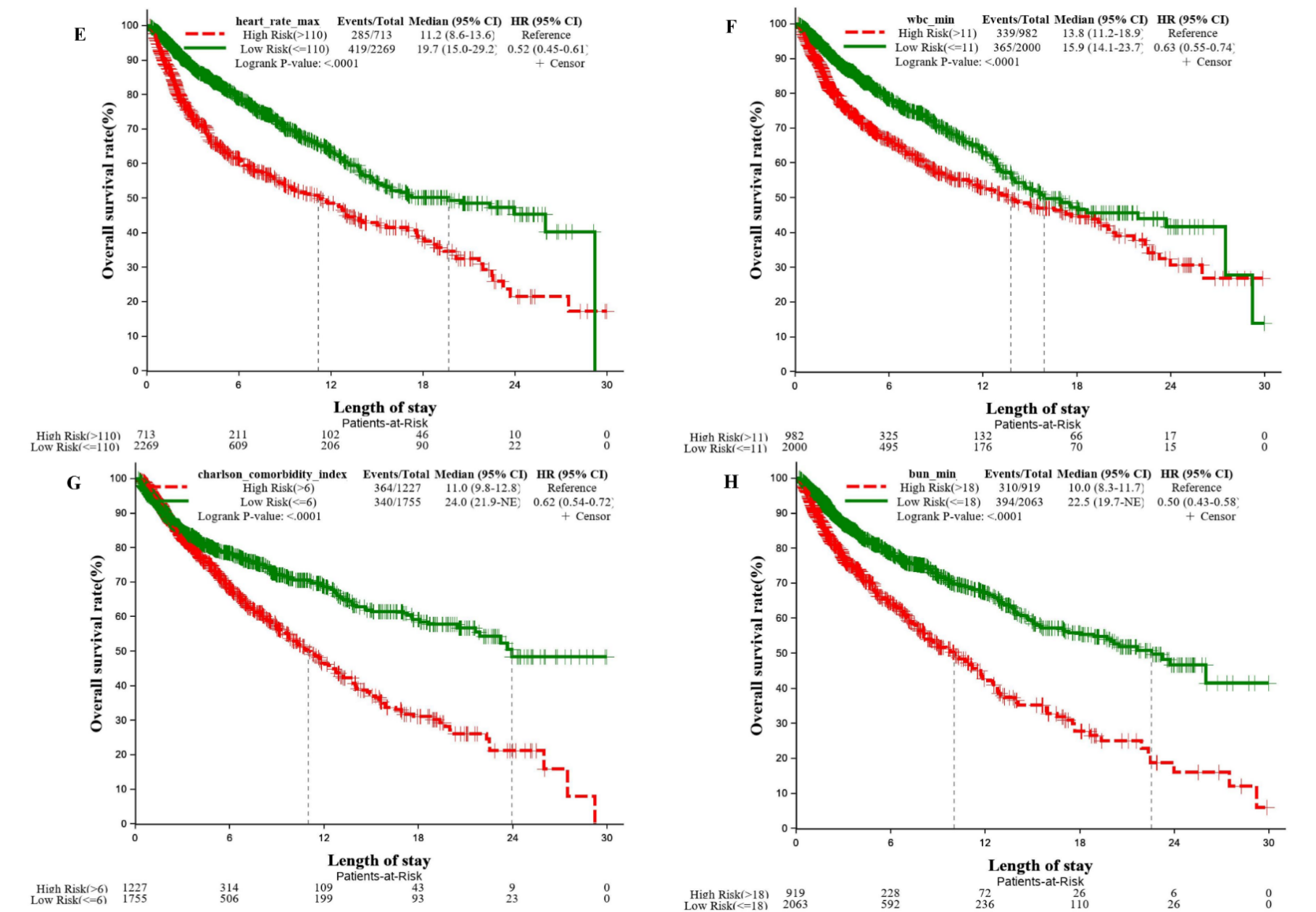


**Figure S6**. Association between selected variables and death risk of stroke patients by Cox proportional hazards models and Kaplan-Meier (KM) survival curves using high risk subgroup as the reference. A: sge; B: sodium_max; C: spo2_mean; D: temperature_max; E: heart_rate_max; F: wbc_min; G: charlson_comorbidity_index; H: bun_min. NE: missing values.


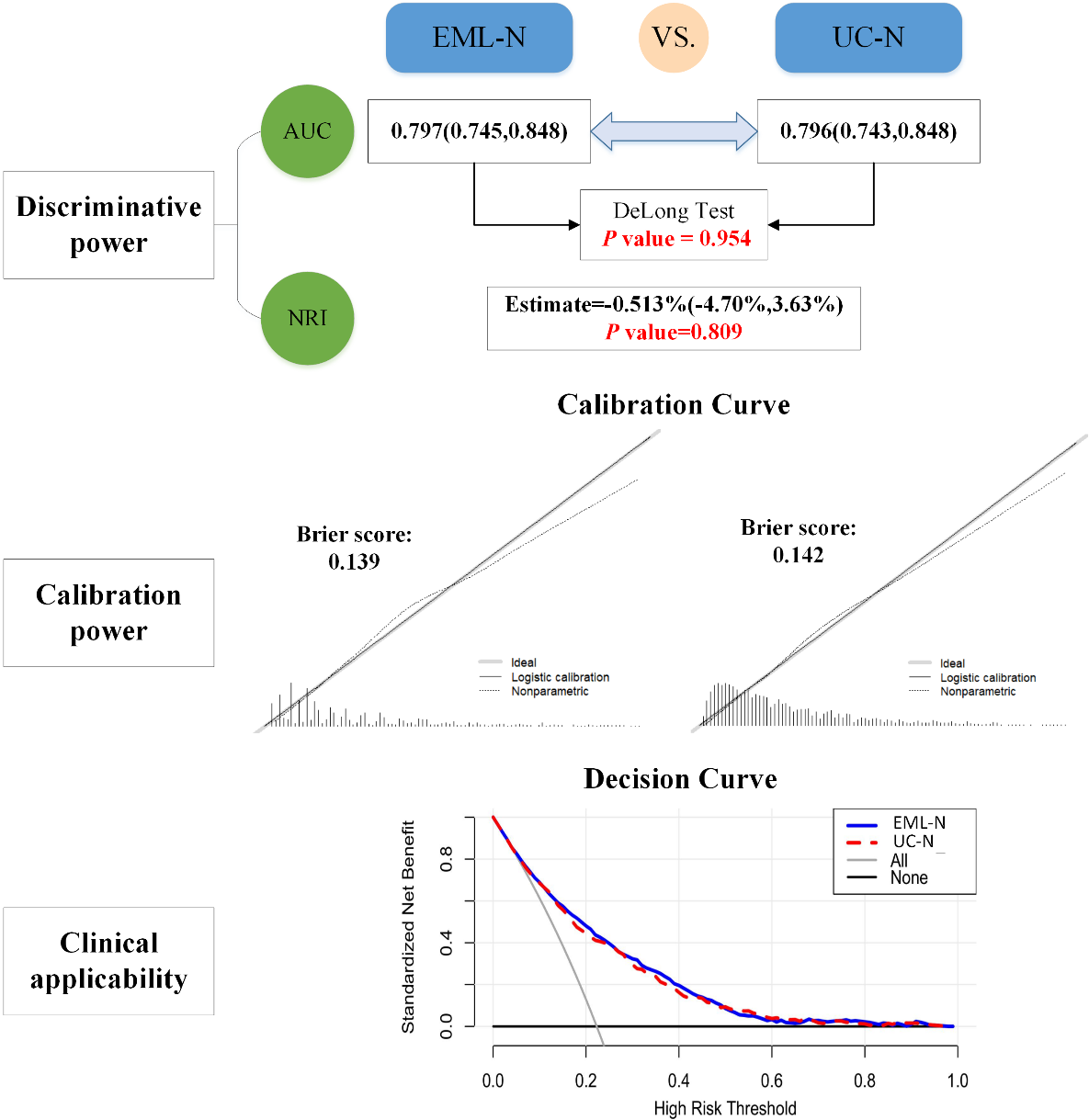


**Figure S7**. Nomograms for predicting 30-day mortality among stroke patients in MIMIC-III datasets. EML-N: explainable machine learning + nomogram; UC-N: unchanged nomogram; AUC: area under the receiver operating characteristics curve; NRI: net reclassification index.


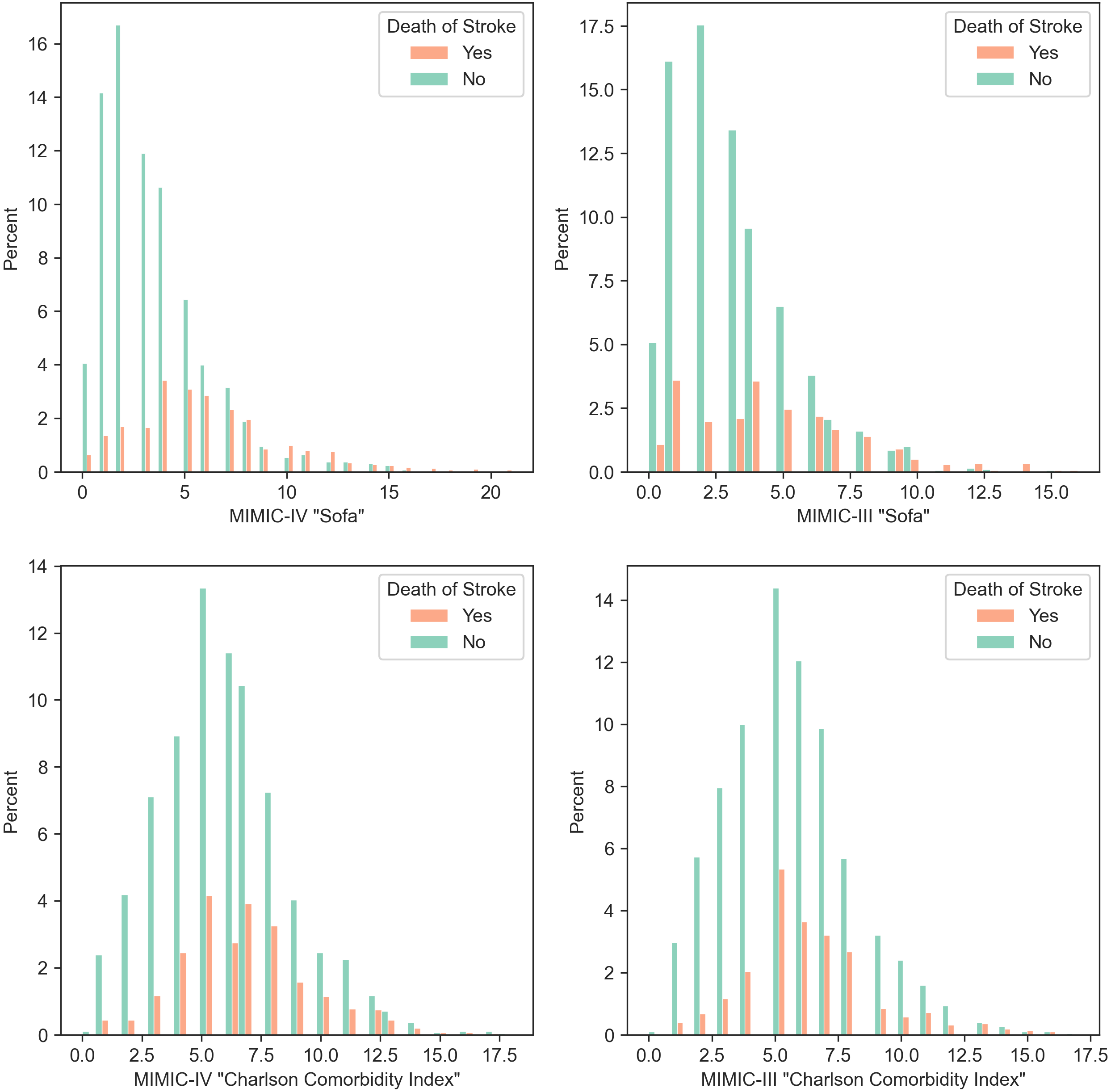


**Figure S8.** The distribution of “sofa” and “charlson comorbidity index” in MIMIC-III and MIMIC-IV datasets.

| **Table S1. All variables extracted from the MIMIC-IV datasets and their explanations in our study.** | | | | | |
| --- | --- | --- | --- | --- | --- |
| **variable** | interpretation | **variable** | interpretation | **variable** | interpretation |
| **insurance** | type of medical insurance | **weight** | - | **dbp min [Minimum Diastolic Blood]** | minimum diastolic blood pressure within 24 hours of icu admission |
| **ethnicity** | - | **heart rate min [Minimum Heart Rate]** | minimum heart rate values within 24 hours of icu admission | **dbp max [Maximum Diastolic blood]** | maximum diastolic blood pressure within 24 hours of icu admission |
| **marital status** | - | **heart rate max [Maximum Heart Rate]** | maximum heart rate within 24 hours of icu admission | **dbp mean [Mean Diastolic Blood Pressure]** | mean diastolic blood pressure within 24 hours of icu admission |
| **gender** | - | **heart rate mean [Mean Heart Rate]** | mean heart rate values within 24 hours of icu admission | **mbp min [Minimum Mean Arterial Pressure]** | minimum mean arterial pressure  within 24 hours of icu admission |
| **diagnosis type** | types of stroke diagnosis | **sbp min [Minimum Systolic Blood Pressure]** | minimum systolic blood pressure within 24 hours of icu admission | **mbp max [Maximum Mean Arterial Pressure]** | maximum mean arterial pressure  within 24 hours of icu admission |
| **los** | length of icu stay | **sbp max [Maximum Systolic Blood Pressure]** | maximum systolic blood pressure within 24 hours of icu admission | **mbp mean [Mean Mean Arterial Pressure]** | mean mean arterial pressure  within 24 hours of icu admission |
| **age** | - | **sbp mean** | mean systolic blood pressure within 24 hours of icu admission | **bun min [Minimum Blood Urea Nitrogen]** | minimum blood urea nitrogen values within 24 hours of icu admission |
| **resp_rate min [Minimum Respiratory Rate]** | minimum respiratory rate within 24 hours of icu admission | **glucose min**  **[Minimum Blood Glucose]** | minimum blood glucose within 24 hours of icu admission | **bun max [Maximum Blood Urea Nitrogen]** | maximum blood urea nitrogen values within 24 hours of icu admission |
| **resp rate max [Maximum Respiratory Rate]** | maximum respiratory rate within 24 hours of icu admission | **glucose max [Maximum Blood glucose]** | maximum blood glucose within 24 hours of icu admission | **hematocrit min [Minimum Hematocrit]** | minimum hematocrit values within 24 hours of icu admission |
| **resp rate mean [Mean Respiratory Rate]** | mean respiratory rate within 24 hours of icu admission | **glucose mean [Mean Blood Glucose]** | mean blood glucose within 24 hours of icu admission | **hematocrit max [Maximum Hematocrit]** | maximum hematocrit values within 24 hours of icu admission |
| **temperature min [Minimum Temperature Rate]** | minimum body temperature within 24 hours of icu admission | **charlson comorbidity index** | a more widely used comorbidity scoring system | **hemoglobin min [Minimum Hemoglobin]** | minimum hemoglobin values within 24 hours of icu admission |
| **temperature max [Maximum Temperature Rate]** | maximum body temperature within 24 hours of icu admission | **sofa** | sepsis-related organ failure assessment: a scoring system to assess the extent and prognosis of organ failure in intensive care patients | **hemoglobin max [Maximum Hemoglobin]** | maximum hemoglobin values within 24 hours of icu admission |
| **temperature mean [Mean Temperature Rate]** | mean body temperature within 24 hours of icu admission | **aniongap min [Minimum Anion Gap]** | minimum anion gap value within 24 hours of icu admission | **platelets min [Minimum platelets]** | minimum platelets values within 24 hours of icu admission |
| **spo2 min [Minimum spo2]** | minimum blood oxygen saturation within 24 hours of icu admission | **aniongap max [Maximum Anion Gap]** | maximum anion gap value within 24 hours of icu admission | **platelets max [Maximum platelets]** | maximum platelets values within 24 hours of icu admission |
| **spo2 max [Maximum spo2]** | maximum blood oxygen saturation within 24 hours of icu admission | **creatinine min [Minimum Creatinine]** | minimum creatinine value within 24 hours of icu admission | **wbc min [Minimum White Blood Cell]** | minimum white blood cell values within 24 hours of icu admission |
| **spo2 mean [Mean spo2]** | mean blood oxygen saturation within 24 hours of icu admission | **creatinine max [Maximum Creatinine]** | maximum creatinine value within 24 hours of icu admission | **wbc max [Maximum White Blood Cell]** | maximum white blood cell values within 24 hours of icu admission |
| **inr min [Minimum INR]** | minimum international normalized ratio values within 24 hours of icu admission | **potassium min [Minimum Potassium]** | minimum potassium value within 24 hours of icu admission | **sodium min [Minimum Sodium]** | minimum sodium value within 24 hours of icu admission |
| **inr max [Maximum INR]** | maximum international normalized ratio values within 24 hours of icu admission | **potassium max [Maximum Potassium]** | maximum potassium value within 24 hours of icu admission | **sodium max [Maximum Sodium]** | maximum sodium value within 24 hours of icu admission |
| **pt min [Minimum PT]** | minimum prothrombin time within 24 hours of icu admission | **chloride min [Minimum Chloride]** | minimum chloride within 24 hours of icu admission | **urine output** | urine output within 24 hours of icu admission |
| **pt max [Maximum PT]** | maximum prothrombin time within 24 hours of icu admission | **chloride max [Maximum Chloride]** | maximum chloride within 24 hours of icu admission |  |  |
| **ptt min [Minimum PTT]** | minimum activated partial thromboplatin time within 24 hours of icu admission | **bicarbonate min [Minimum Bicarbonate]** | minimum bicarbonate within 24 hours of icu admission |  |  |
| **ptt max [Maximum PTT]** | maximum activated partial thromboplatin time within 24 hours of icu admission | **bicarbonate max [Maximum Bicarbonate]** | maximum bicarbonate within 24 hours of icu admission |  |  |

**Table S2. Comparison of all variables between survived and dead patients.**

|  | **Death of Stroke** | |  | |
| --- | --- | --- | --- | --- |
| **Variable** | No (N=2278) | Yes (N=704) | Total (N=2982) | ***P*** **value** |
| **insurance**, n (%) |  |  |  | 0.0001^1^ |
| Medicaid | 126 (5.5%) | 33 (4.7%) | 159 (5.3%) |  |
| Medicare | 915 (40.2%) | 346 (49.1%) | 1261 (42.3%) |  |
| Other | 1237 (54.3%) | 325 (46.2%) | 1562 (52.4%) |  |
| **ethnicity**, n (%) |  |  |  | <.0001^1^ |
| American Indian/Alaska | 8 (0.4%) | 0 (0.0%) | 8 (0.3%) |  |
| Asian | 67 (2.9%) | 24 (3.4%) | 91 (3.1%) |  |
| Black/Africa American | 218 (9.6%) | 55 (7.8%) | 273 (9.2%) |  |
| Hispanic/Latino | 77 (3.4%) | 22 (3.1%) | 99 (3.3%) |  |
| Other | 133 (5.8%) | 29 (4.1%) | 162 (5.4%) |  |
| Unable to obtain | 27 (1.2%) | 24 (3.4%) | 51 (1.7%) |  |
| Unknown | 279 (12.2%) | 202 (28.7%) | 481 (16.1%) |  |
| White | 1469 (64.5%) | 348 (49.4%) | 1817 (60.9%) |  |
| **marital status**, n (%) |  |  |  | 0.0120^1^ |
| Divorced | 176 (8.6%) | 35 (7.1%) | 211 (8.3%) |  |
| Married | 1099 (53.6%) | 265 (54.0%) | 1364 (53.7%) |  |
| Single | 522 (25.5%) | 106 (21.6%) | 628 (24.7%) |  |
| Windowed | 252 (12.3%) | 85 (17.3%) | 337 (13.3%) |  |
| Missing | 229 | 213 | 442 |  |
| **gender**, n (%) |  |  |  | 0.2802^1^ |
| F | 1086 (47.7%) | 352 (50.0%) | 1438 (48.2%) |  |
| M | 1192 (52.3%) | 352 (50.0%) | 1544 (51.8%) |  |
| **diagnosis type**, n (%) |  |  |  | <.0001^1^ |
| IH | 891 (39.1%) | 350 (49.7%) | 1241 (41.6%) |  |
| IS | 855 (37.5%) | 227 (32.2%) | 1082 (36.3%) |  |
| SH | 392 (17.2%) | 125 (17.8%) | 517 (17.3%) |  |
| TIA | 140 (6.1%) | 2 (0.3%) | 142 (4.8%) |  |
| **los** |  |  |  | 0.0150^2^ |
| N | 2278 | 704 | 2982 |  |
| Mean (SD) | 5.1 (5.40) | 4.7 (4.95) | 5.0 (5.30) |  |
| Median | 3.0 | 2.7 | 2.9 |  |
| Range | 0.2, 30.0 | 0.2, 29.2 | 0.2, 30.0 |  |
| **age** |  |  |  | <.0001^2^ |
| N | 2278 | 704 | 2982 |  |
| Mean (SD) | 65.3 (15.27) | 70.6 (13.27) | 66.6 (14.99) |  |
| Median | 66.7 | 72.6 | 68.3 |  |
| Range | 18.0, 89.0 | 22.8, 89.0 | 18.0, 89.0 |  |
| **weight** |  |  |  | 0.0002^2^ |
| N | 2278 | 704 | 2982 |  |
| Mean (SD) | 80.5 (21.72) | 76.9 (21.73) | 79.7 (21.77) |  |
| Median | 78.0 | 75.0 | 77.2 |  |
| Range | 1.0, 206.7 | 1.0, 212.3 | 1.0, 212.3 |  |
| **minimum heart rate** |  |  |  | 0.0226^2^ |
| N | 2278 | 704 | 2982 |  |
| Mean (SD) | 64.4 (12.26) | 65.4 (17.51) | 64.7 (13.68) |  |
| Median | 63.0 | 65.0 | 64.0 |  |
| Range | 20.0, 113.0 | 7.0, 128.0 | 7.0, 128.0 |  |
| **maximum heart rate** |  |  |  | <.0001^2^ |
| N | 2278 | 704 | 2982 |  |
| Mean (SD) | 96.5 (17.82) | 107.3 (22.62) | 99.0 (19.60) |  |
| Median | 94.0 | 105.0 | 97.0 |  |
| Range | 55.0, 190.0 | 56.0, 197.0 | 55.0, 197.0 |  |
| **mean heart rate** |  |  |  | <.0001^2^ |
| N | 2278 | 704 | 2982 |  |
| Mean (SD) | 78.2 (13.30) | 84.0 (16.13) | 79.6 (14.24) |  |
| Median | 77.0 | 82.8 | 78.3 |  |
| Range | 44.8, 128.9 | 42.6, 149.0 | 42.6, 149.0 |  |
| **minimum sbp** |  |  |  | <.0001^2^ |
| N | 2274 | 701 | 2975 |  |
| Mean (SD) | 103.3 (16.73) | 95.8 (24.02) | 101.5 (18.97) |  |
| Median | 103.0 | 97.0 | 102.0 |  |
| Range | 2.0, 169.0 | 16.0, 181.0 | 2.0, 181.0 |  |
| **maximum sbp** |  |  |  | <.0001^2^ |
| N | 2274 | 701 | 2975 |  |
| Mean (SD) | 160.2 (22.17) | 164.0 (25.05) | 161.1 (22.93) |  |
| Median | 158.3 | 163.0 | 160.0 |  |
| Range | 78.0, 352.0 | 77.0, 257.0 | 77.0, 352.0 |  |
| **mean sbp** |  |  |  | 0.0020^2^ |
| N | 2274 | 701 | 2975 |  |
| Mean (SD) | 130.6 (15.78) | 127.9 (18.06) | 129.9 (16.39) |  |
| Median | 130.0 | 128.7 | 129.8 |  |
| Range | 75.0, 195.3 | 52.6, 197.2 | 52.6, 197.2 |  |
| **minimum dbp** |  |  |  | <.0001^2^ |
| N | 2274 | 701 | 2975 |  |
| Mean (SD) | 51.4 (11.37) | 46.9 (13.08) | 50.4 (11.95) |  |
| Median | 51.0 | 47.0 | 50.0 |  |
| Range | 10.0, 93.0 | 7.0, 114.0 | 7.0, 114.0 |  |
| **maximum dbp** |  |  |  | 0.0003^2^ |
| N | 2274 | 701 | 2975 |  |
| Mean (SD) | 93.9 (19.45) | 91.6 (21.49) | 93.3 (19.97) |  |
| Median | 92.0 | 89.0 | 91.0 |  |
| Range | 47.0, 273.0 | 36.0, 181.0 | 36.0, 273.0 |  |
| **mean dbp** |  |  |  | <.0001^2^ |
| N | 2274 | 701 | 2975 |  |
| Mean (SD) | 68.3 (11.65) | 64.9 (11.53) | 67.5 (11.71) |  |
| Median | 67.6 | 63.9 | 66.8 |  |
| Range | 37.8, 117.1 | 29.2, 129.6 | 29.2, 129.6 |  |
| **minimum mbp** |  |  |  | <.0001^2^ |
| N | 2277 | 703 | 2980 |  |
| Mean (SD) | 65.6 (14.19) | 59.4 (16.94) | 64.1 (15.11) |  |
| Median | 66.0 | 61.0 | 65.0 |  |
| Range | 1.0, 113.0 | 2.0, 133.0 | 1.0, 133.0 |  |
| **maximum mbp** |  |  |  | 0.1337^2^ |
| N | 2277 | 703 | 2980 |  |
| Mean (SD) | 111.6 (20.27) | 111.9 (25.70) | 111.7 (21.67) |  |
| Median | 109.0 | 108.0 | 109.0 |  |
| Range | 68.0, 276.0 | 47.0, 290.0 | 47.0, 290.0 |  |
| **mean mbp** |  |  |  | <.0001^2^ |
| N | 2277 | 703 | 2980 |  |
| Mean (SD) | 85.5 (11.09) | 82.2 (11.17) | 84.7 (11.19) |  |
| Median | 84.9 | 82.0 | 84.2 |  |
| Range | 57.6, 139.9 | 38.5, 133.1 | 38.5, 139.9 |  |
| **minimum resp rate** |  |  |  | <.0001^2^ |
| N | 2274 | 704 | 2978 |  |
| Mean (SD) | 12.2 (2.95) | 12.9 (3.83) | 12.3 (3.19) |  |
| Median | 12.0 | 13.0 | 12.0 |  |
| Range | 1.0, 27.5 | 2.0, 28.0 | 1.0, 28.0 |  |
| **maximum resp rate** |  |  |  | 0.0002^2^ |
| N | 2274 | 704 | 2978 |  |
| Mean (SD) | 26.3 (5.65) | 27.3 (6.22) | 26.5 (5.81) |  |
| Median | 25.0 | 26.0 | 26.0 |  |
| Range | 15.0, 67.0 | 12.0, 57.0 | 12.0, 67.0 |  |
| **mean resp rate** |  |  |  | <.0001^2^ |
| N | 2274 | 704 | 2978 |  |
| Mean (SD) | 18.2 (2.99) | 19.6 (3.66) | 18.5 (3.21) |  |
| Median | 17.9 | 19.0 | 18.0 |  |
| Range | 11.0, 38.8 | 9.1, 32.7 | 9.1, 38.8 |  |
| **minimum temperature** |  |  |  | 0.0319^2^ |
| N | 2249 | 690 | 2939 |  |
| Mean (SD) | 36.5 (0.55) | 36.3 (1.02) | 36.4 (0.69) |  |
| Median | 36.6 | 36.5 | 36.6 |  |
| Range | 30.2, 39.8 | 28.6, 39.2 | 28.6, 39.8 |  |
| **maximum temperature** |  |  |  | <.0001^2^ |
| N | 2249 | 690 | 2939 |  |
| Mean (SD) | 37.4 (0.59) | 37.7 (1.06) | 37.5 (0.74) |  |
| Median | 37.3 | 37.7 | 37.3 |  |
| Range | 33.8, 40.2 | 31.8, 41.3 | 31.8, 41.3 |  |
| **mean temperature** |  |  |  | <.0001^2^ |
| N | 2249 | 690 | 2939 |  |
| Mean (SD) | 36.9 (0.41) | 37.0 (0.85) | 37.0 (0.55) |  |
| Median | 36.9 | 37.1 | 36.9 |  |
| Range | 33.0, 40.0 | 31.0, 39.7 | 31.0, 40.0 |  |
| **minimum spo2** |  |  |  | 0.0112^2^ |
| N | 2278 | 702 | 2980 |  |
| Mean (SD) | 92.9 (5.24) | 89.7 (14.15) | 92.2 (8.36) |  |
| Median | 93.0 | 94.0 | 94.0 |  |
| Range | 1.0, 100.0 | 7.0, 100.0 | 1.0, 100.0 |  |
| **maximum spo2** |  |  |  | <.0001^2^ |
| N | 2278 | 702 | 2980 |  |
| Mean (SD) | 99.4 (1.00) | 99.7 (0.97) | 99.5 (1.00) |  |
| Median | 100.0 | 100.0 | 100.0 |  |
| Range | 94.0, 100.0 | 90.0, 100.0 | 90.0, 100.0 |  |
| **mean spo2** |  |  |  | <.0001^2^ |
| N | 2278 | 702 | 2980 |  |
| Mean (SD) | 97.1 (1.73) | 97.0 (3.68) | 97.0 (2.34) |  |
| Median | 97.1 | 98.0 | 97.3 |  |
| Range | 79.6, 100.0 | 67.0, 100.0 | 67.0, 100.0 |  |
| **minimum glucose** |  |  |  | <.0001^2^ |
| N | 2260 | 633 | 2893 |  |
| Mean (SD) | 110.8 (28.76) | 129.2 (42.54) | 114.8 (33.16) |  |
| Median | 105.0 | 123.0 | 109.0 |  |
| Range | 32.0, 283.0 | 20.0, 365.0 | 20.0, 365.0 |  |
| **maximum glucose** |  |  |  | <.0001^2^ |
| N | 2260 | 633 | 2893 |  |
| Mean (SD) | 604.8 (21031.86) | 202.4 (89.10) | 516.7 (18588.96) |  |
| Median | 145.0 | 178.0 | 150.0 |  |
| Range | 72.0, 999999.0 | 75.0, 708.0 | 72.0, 999999.0 |  |
| **mean glucose** |  |  |  | <.0001^2^ |
| N | 2260 | 633 | 2893 |  |
| Mean (SD) | 197.4 (3004.78) | 161.0 (49.52) | 189.4 (2655.80) |  |
| Median | 124.5 | 150.0 | 129.9 |  |
| Range | 68.5, 142966.9 | 72.3, 384.5 | 68.5, 142966.9 |  |
| **charlson comorbidity index** |  |  |  | <.0001^2^ |
| N | 2278 | 704 | 2982 |  |
| Mean (SD) | 5.9 (2.68) | 6.8 (2.72) | 6.1 (2.71) |  |
| Median | 6.0 | 7.0 | 6.0 |  |
| Range | 0.0, 18.0 | 1.0, 17.0 | 0.0, 18.0 |  |
| **sofa** |  |  |  | <.0001^2^ |
| N | 2278 | 704 | 2982 |  |
| Mean (SD) | 3.4 (2.62) | 6.1 (3.69) | 4.0 (3.12) |  |
| Median | 3.0 | 5.5 | 3.0 |  |
| Range | 0.0, 17.0 | 0.0, 21.0 | 0.0, 21.0 |  |
| **minimum anion gap** |  |  |  | <.0001^2^ |
| N | 2247 | 667 | 2914 |  |
| Mean (SD) | 13.4 (2.77) | 14.5 (3.81) | 13.7 (3.08) |  |
| Median | 13.0 | 14.0 | 13.0 |  |
| Range | -3.0, 29.0 | 3.0, 38.0 | -3.0, 38.0 |  |
| **maximum aniongap** |  |  |  | <.0001^2^ |
| N | 2247 | 667 | 2914 |  |
| Mean (SD) | 15.7 (3.36) | 17.7 (4.77) | 16.1 (3.82) |  |
| Median | 15.0 | 17.0 | 16.0 |  |
| Range | 6.0, 43.0 | 9.0, 49.0 | 6.0, 49.0 |  |
| **minimum creatinine** |  |  |  | <.0001^2^ |
| N | 2256 | 697 | 2953 |  |
| Mean (SD) | 1.0 (0.85) | 1.2 (1.06) | 1.0 (0.91) |  |
| Median | 0.8 | 0.9 | 0.8 |  |
| Range | 0.1, 18.5 | 0.1, 10.9 | 0.1, 18.5 |  |
| **maximum creatinine** |  |  |  | <.0001^2^ |
| N | 2256 | 697 | 2953 |  |
| Mean (SD) | 1.1 (0.96) | 1.4 (1.27) | 1.2 (1.05) |  |
| Median | 0.9 | 1.0 | 0.9 |  |
| Range | 0.2, 19.7 | 0.3, 11.5 | 0.2, 19.7 |  |
| **minimum bun** |  |  |  | <.0001^2^ |
| N | 2258 | 698 | 2956 |  |
| Mean (SD) | 16.6 (10.69) | 22.5 (18.20) | 17.9 (13.11) |  |
| Median | 14.0 | 18.0 | 15.0 |  |
| Range | 2.0, 180.0 | 3.0, 181.0 | 2.0, 181.0 |  |
| **maximum bun** |  |  |  | <.0001^2^ |
| N | 2258 | 698 | 2956 |  |
| Mean (SD) | 19.5 (11.84) | 26.6 (21.47) | 21.2 (15.00) |  |
| Median | 17.0 | 20.5 | 18.0 |  |
| Range | 3.0, 190.0 | 4.0, 211.0 | 3.0, 211.0 |  |
| **minimum hematocrit** |  |  |  | <.0001^2^ |
| N | 2256 | 695 | 2951 |  |
| Mean (SD) | 35.6 (6.02) | 34.1 (6.52) | 35.3 (6.17) |  |
| Median | 36.2 | 34.6 | 35.8 |  |
| Range | 12.1, 54.6 | 13.5, 56.7 | 12.1, 56.7 |  |
| **maximum hematocrit** |  |  |  | 0.0174^2^ |
| N | 2256 | 695 | 2951 |  |
| Mean (SD) | 38.6 (5.47) | 38.0 (6.30) | 38.5 (5.68) |  |
| Median | 38.8 | 38.2 | 38.8 |  |
| Range | 19.4, 60.1 | 20.8, 64.6 | 19.4, 64.6 |  |
| **minimum hemoglobin** |  |  |  | <.0001^2^ |
| N | 2256 | 694 | 2950 |  |
| Mean (SD) | 11.9 (2.10) | 11.4 (2.27) | 11.8 (2.15) |  |
| Median | 12.1 | 11.5 | 12.0 |  |
| Range | 3.8, 18.6 | 4.4, 18.4 | 3.8, 18.6 |  |
| **maximum hemoglobin** |  |  |  | 0.0003^2^ |
| N | 2256 | 694 | 2950 |  |
| Mean (SD) | 12.9 (1.94) | 12.6 (2.20) | 12.8 (2.01) |  |
| Median | 13.0 | 12.7 | 13.0 |  |
| Range | 6.1, 19.9 | 6.2, 20.3 | 6.1, 20.3 |  |
| **minimum platelets** |  |  |  | <.0001^2^ |
| N | 2255 | 693 | 2948 |  |
| Mean (SD) | 211.3 (80.96) | 196.3 (93.38) | 207.8 (84.27) |  |
| Median | 202.0 | 189.0 | 199.0 |  |
| Range | 16.0, 1269.0 | 6.0, 817.0 | 6.0, 1269.0 |  |
| **maximum platelets** |  |  |  | 0.0045^2^ |
| N | 2255 | 693 | 2948 |  |
| Mean (SD) | 236.4 (88.62) | 230.1 (106.07) | 235.0 (93.03) |  |
| Median | 223.0 | 219.0 | 222.0 |  |
| Range | 34.0, 1302.0 | 28.0, 932.0 | 28.0, 1302.0 |  |
| **minimum wbc** |  |  |  | <.0001^2^ |
| N | 2256 | 695 | 2951 |  |
| Mean (SD) | 9.7 (4.48) | 12.0 (9.06) | 10.3 (5.97) |  |
| Median | 9.1 | 10.9 | 9.4 |  |
| Range | 0.3, 121.0 | 0.1, 199.3 | 0.1, 199.3 |  |
| **maximum wbc** |  |  |  | <.0001^2^ |
| N | 2256 | 695 | 2951 |  |
| Mean (SD) | 12.1 (8.41) | 15.5 (10.94) | 12.9 (9.18) |  |
| Median | 10.9 | 14.0 | 11.6 |  |
| Range | 0.6, 328.4 | 0.1, 235.0 | 0.1, 328.4 |  |
| **minimum inr** |  |  |  | <.0001^2^ |
| N | 2167 | 685 | 2852 |  |
| Mean (SD) | 1.1 (0.22) | 1.3 (1.17) | 1.2 (0.61) |  |
| Median | 1.1 | 1.1 | 1.1 |  |
| Range | 0.8, 3.8 | 0.6, 26.7 | 0.6, 26.7 |  |
| **maximum inr** |  |  |  | <.0001^2^ |
| N | 2167 | 685 | 2852 |  |
| Mean (SD) | 1.3 (0.51) | 1.5 (1.56) | 1.3 (0.89) |  |
| Median | 1.1 | 1.2 | 1.1 |  |
| Range | 0.9, 12.1 | 0.9, 26.7 | 0.9, 26.7 |  |
| **minimum pt** |  |  |  | <.0001^2^ |
| N | 2167 | 685 | 2852 |  |
| Mean (SD) | 12.5 (2.38) | 13.8 (7.89) | 12.9 (4.42) |  |
| Median | 12.1 | 12.6 | 12.2 |  |
| Range | 8.2, 40.4 | 8.3, 147.0 | 8.2, 147.0 |  |
| **maximum pt** |  |  |  | <.0001^2^ |
| N | 2167 | 685 | 2852 |  |
| Mean (SD) | 13.8 (5.24) | 16.5 (12.90) | 14.4 (7.88) |  |
| Median | 12.5 | 13.5 | 12.7 |  |
| Range | 9.4, 135.7 | 9.5, 150.0 | 9.4, 150.0 |  |
| **minimum ptt** |  |  |  | 0.4969^2^ |
| N | 2165 | 684 | 2849 |  |
| Mean (SD) | 28.0 (7.00) | 28.4 (9.74) | 28.1 (7.75) |  |
| Median | 27.0 | 26.8 | 26.9 |  |
| Range | 16.8, 150.0 | 17.8, 150.0 | 16.8, 150.0 |  |
| **maximum ptt** |  |  |  | 0.1176^2^ |
| N | 2165 | 684 | 2849 |  |
| Mean (SD) | 35.0 (20.78) | 38.7 (27.80) | 35.9 (22.71) |  |
| Median | 29.0 | 29.8 | 29.2 |  |
| Range | 18.1, 150.0 | 17.8, 150.0 | 17.8, 150.0 |  |
| **Minimum potassium** |  |  |  | 0.0595^2^ |
| N | 2249 | 669 | 2918 |  |
| Mean (SD) | 3.8 (0.46) | 3.8 (0.62) | 3.8 (0.50) |  |
| Median | 3.8 | 3.8 | 3.8 |  |
| Range | 1.7, 6.5 | 1.4, 8.7 | 1.4, 8.7 |  |
| **maximum potassium** |  |  |  | <.0001^2^ |
| N | 2249 | 669 | 2918 |  |
| Mean (SD) | 4.3 (0.70) | 4.5 (0.85) | 4.3 (0.74) |  |
| Median | 4.1 | 4.3 | 4.2 |  |
| Range | 2.5, 9.4 | 2.8, 9.0 | 2.5, 9.4 |  |
| **minimum chloride** |  |  |  | 0.0657^2^ |
| N | 2248 | 669 | 2917 |  |
| Mean (SD) | 102.8 (4.75) | 102.3 (6.00) | 102.7 (5.07) |  |
| Median | 103.0 | 103.0 | 103.0 |  |
| Range | 63.0, 134.0 | 76.0, 130.0 | 63.0, 134.0 |  |
| **maximum chloride** |  |  |  | <.0001^2^ |
| N | 2248 | 669 | 2917 |  |
| Mean (SD) | 105.8 (4.88) | 107.8 (7.64) | 106.3 (5.69) |  |
| Median | 106.0 | 107.0 | 106.0 |  |
| Range | 83.0, 140.0 | 88.0, 140.0 | 83.0, 140.0 |  |
| **minimum bicarbonate** |  |  |  | <.0001^2^ |
| N | 2248 | 667 | 2915 |  |
| Mean (SD) | 22.7 (3.38) | 21.1 (4.57) | 22.3 (3.74) |  |
| Median | 23.0 | 21.0 | 23.0 |  |
| Range | 9.0, 35.0 | 3.0, 40.0 | 3.0, 40.0 |  |
| **maximum bicarbonate** |  |  |  | <.0001^2^ |
| N | 2248 | 667 | 2915 |  |
| Mean (SD) | 24.6 (3.09) | 23.7 (3.95) | 24.4 (3.33) |  |
| Median | 25.0 | 24.0 | 24.0 |  |
| Range | 14.0, 44.0 | 11.0, 45.0 | 11.0, 45.0 |  |
| **minimum sodium** |  |  |  | 0.1399^2^ |
| N | 2249 | 669 | 2918 |  |
| Mean (SD) | 138.2 (4.04) | 137.9 (5.41) | 138.1 (4.39) |  |
| Median | 138.0 | 138.0 | 138.0 |  |
| Range | 93.0, 170.0 | 114.0, 160.0 | 93.0, 170.0 |  |
| **maximum sodium** |  |  |  | <.0001^2^ |
| N | 2249 | 669 | 2918 |  |
| Mean (SD) | 140.6 (3.88) | 142.9 (6.79) | 141.1 (4.81) |  |
| Median | 141.0 | 142.0 | 141.0 |  |
| Range | 119.0, 174.0 | 125.0, 179.0 | 119.0, 179.0 |  |
| **urine output** |  |  |  | 0.0003^2^ |
| N | 2278 | 704 | 2982 |  |
| Mean (SD) | 1829.6 (1035.34) | 1810.9 (1437.26) | 1825.2 (1142.80) |  |
| Median | 1675.0 | 1466.5 | 1617.5 |  |
| Range | -1305.0, 8235.0 | 0.0, 9940.0 | -1305.0, 9940.0 |  |
| ^1^Chi-Square p-value; ^2^Kruskal-Wallis p-value; | | | | |

| **Table S3**. Covariance diagnosis for 10 variables selected. | |
| --- | --- |
| **Variable** | Variance inflation factor (VIF) |
| Sofa | 1.323 |
| Minimum Glucose | 1.058 |
| Maximum Sodium | 1.068 |
| Age | 1.550 |
| Mean Spo2 | 1.063 |
| Maximum Temperature | 1.145 |
| Maximum Heart Rate | 1.139 |
| Minimum Bun | 1.310 |
| Minimum Wbc | 1.042 |
| Charlson Comorbidity Index | 1.705 |

| **Table S4.** Comparison of 10 selected categorical variables between survived and dead patients. | | | | |
| --- | --- | --- | --- | --- |
|  | **Death of Stroke** | |  | |
| **Variable** | No (N=2278) | Yes (N=704) | Total (N=2982) | P-value |
| **sofa**, n (%) |  |  |  | <.001^1^ |
| 0 (High Risk) | 565 (24.8%) | 444 (63.1%) | 1009 (33.8%) |  |
| 1 (Low Risk) | 1713 (75.2%) | 260 (36.9%) | 1973 (66.2%) |  |
| **minimum glucose**, n (%) |  |  |  | <.001^1^ |
| 0 (High Risk) | 963 (42.3%) | 404 (57.4%) | 1367 (45.8%) |  |
| 1 (Low Risk) | 1315 (57.7%) | 300 (42.6%) | 1615 (54.2%) |  |
| **maximum sodium**, n (%) |  |  |  | <.001^1^ |
| 0 (High Risk) | 167 (7.3%) | 199 (28.3%) | 366 (12.3%) |  |
| 1 (Low Risk) | 2111 (92.7%) | 505 (71.7%) | 2616 (87.7%) |  |
| **age**, n (%) |  |  |  | <.001^1^ |
| 0 (High Risk) | 951 (41.7%) | 401 (57.0%) | 1352 (45.3%) |  |
| 1 (Low Risk) | 1327 (58.3%) | 303 (43.0%) | 1630 (54.7%) |  |
| **mean spo2**, n (%) |  |  |  | <.001^1^ |
| 0 (High Risk) | 25 (1.1%) | 68 (9.7%) | 93 (3.1%) |  |
| 1 (Low Risk) | 2253 (98.9%) | 636 (90.3%) | 2889 (96.9%) |  |
| **maximum temperature**, n (%) |  |  |  | <.001^1^ |
| 0 (High Risk) | 608 (26.7%) | 402 (57.1%) | 1010 (33.9%) |  |
| 1 (Low Risk) | 1670 (73.3%) | 302 (42.9%) | 1972 (66.1%) |  |
| **maximum heart rate**, n (%) |  |  |  | <.001^1^ |
| 0 (High Risk) | 428 (18.8%) | 285 (40.5%) | 713 (23.9%) |  |
| 1 (Low Risk) | 1850 (81.2%) | 419 (59.5%) | 2269 (76.1%) |  |
| **minimum bun**, n (%) |  |  |  | <.001^1^ |
| 0 (High Risk) | 609 (26.7%) | 310 (44.0%) | 919 (30.8%) |  |
| 1 (Low Risk) | 1669 (73.3%) | 394 (56.0%) | 2063 (69.2%) |  |
| **minimum wbc**, n (%) |  |  |  | <.001^1^ |
| 0 (High Risk) | 643 (28.2%) | 339 (48.2%) | 982 (32.9%) |  |
| 1 (Low Risk) | 1635 (71.8%) | 365 (51.8%) | 2000 (67.1%) |  |
| **charlson comorbidity index**, n (%) |  |  |  | <.001^1^ |
| 0 (High Risk) | 863 (37.9%) | 364 (51.7%) | 1227 (41.1%) |  |
| 1 (Low Risk) | 1415 (62.1%) | 340 (48.3%) | 1755 (58.9%) |  |
| ^1^Chi-Square p-value; | | | | |
